# Supplementary material for: The genetic landscape of human functional brain connectivity
Source: Nat Commun. 2026 Feb 24;17:3120. doi: 10.1038/s41467-026-69442-9 (PMC13043896; doi:10.1038/s41467-026-69442-9)
Supplement: Supplementary file 1 — Supplementary Information [file 41467_2026_69442_MOESM1_ESM.pdf]

**Supplementary Notes and Figures for**

**The genetic landscape of human functional brain connectivity**

References of figures and tables for each supplementary note are labelled Supplementary Note Figure/Table.


**Authors**

Bernardo de APC Maciel[1], Marijn Schipper[1], Cato Romero[2], Christiaan de Leeuw[1], Koen Helwegen[1], Danielle Posthuma[1,3], Jeanne E. Savage[1], Martijn P. van den Heuvel*[1,2]

[1] Department of Complex Trait Genetics, Center for Neurogenomics and Cognitive Research, Amsterdam Neuroscience, Vrije Universiteit Amsterdam, Amsterdam, the Netherlands

[2] Department of Child and Adolescent Psychiatry and Psychology, Section Complex Trait Genetics, Amsterdam Neuroscience, Vrije Universiteit Medical Center, Amsterdam UMC, Amsterdam, the Netherlands

[3] Department of Clinical Genetics, Section Complex Trait Genetics, Amsterdam Neuroscience, Vrije Universiteit Medical Center, Amsterdam University Medical Centre, Amsterdam 1081 HZ, the Netherlands

* Corresponding author: martijn.vanden.heuvel@vu.nl

**Table of Contents**


Supplementary Methods ........ 2
- Subject Exclusions & Quality Control ........ 2
- Subnetwork Definition ........ 3
- Subnetwork Enrichment Analysis ........ 3
- Gene effect extent ........ 4
- LD-aware and LD-agnostic null models ........ 5
- Null distribution generation procedure ........ 6

Supplementary Note 1 - Sample Demographics ........ 7

Supplementary Note 2 - Subnetwork Enrichment Analyses ........ 10

Supplementary Note 3 - Reliability Analysis ........ 12
- Relationship between heritability and measurement reliability ........ 12
- Global Signal Regression Effect ........ 12

Supplementary Note 4 - Validation of overlapping loci ........ 17

Supplementary Note 5 - Comparison of phenotypic definitions ........ 20
- Comparison with edge-wise ICA-derived functional connectivity ........ 20
- Comparison with average resting-state network functional connectivity ........ 20

Supplementary Note 6 - Genetic overlap analyses of functional connectivity and disorder 23
- GWAS and Gene-sets for disorder ........ 23
- LDSC analysis ........ 23
- Mendelian Randomisation ........ 24
- Disorder gene-set effect extent comparison ........ 24

References ........ 30

List of Supplementary Note Tables and Figures ........ 32

Supplementary Figures ........ 33
- Supplementary Figure 1. Visual summary of methods and results. ........ 33
- Supplementary Figure 2. QQ Plots for association of locus across the brain. ........ 34
- Supplementary Figure 3. Effect extent of disease genes in the functional connectome. 35

Supplementary Figure 4. Gene-set enrichment testing for the set of replicated genes (5 genes). ........ 36
- Supplementary Figure 5. Protein-Protein Interaction Network. ........ 37
- Supplementary Figure 6. Gene-set enrichment testing for complete set of replicated genes. ........ 38
- Supplementary Figure 7. Volcano plots of genetic correlations between edge GWAS and disorder GWAS. ........ 40
- Supplementary Figure 8. Tissue Expression heatmap of replicated genes. ........ 41
- Supplementary Figure 9. Lifetime expression heatmap of replicated genes. ........ 41

**Supplementary Methods**

*Subject Exclusions & Quality Control*

Data were derived from the UK Biobank (UKB), a population-based cohort sample of approximately 500,000 adults in the UK. Participants were invited for an initial in-person visit to a study assessment centre in 2006-2010, during which numerous physical measurements and surveys were administered, and blood samples were collected for genotyping. Participant data was linked to medical records via the National Health Services, and a subset of participants also completed MRI scans and additional follow-up data collection in subsequent years. All participants provided written informed consent, those who revoked their consent at the time of the main analysis (January 2023) were removed from subsequent students. The UKB received ethical approval from the National Research Ethics Service Committee North West-Haydock (reference 11/NW/0382), and all study procedures were in accordance with the World Medical Association for medical research. Access to the UK Biobank data was obtained under application number 16406.

In the full UKB sample, 481 subjects with sex aneuploidy, 370 with discordant reported and chromosomal sex, and 195 with high degrees of relatedness were excluded from further analyses. Next, on the subset of subjects with one imaging visit available ($n$ = 36,969), related subjects were excluded. Subjects with high levels of kinship (KING coefficient>0.4) and the most inferred relatives were removed until no two subjects were reported to be third-degree relatives (or closer), accounting for a total of 5,241 exclusions. Population stratification was controlled by correcting for principal genomic components calculated with FLASHPCA2.[1] Principal components from the 1000 Genomes reference populations[2] were projected onto the called genotypes available in the UKB data and all 2,034 subjects for whom the projected scores were the furthest to the average score of Europeans (i.e. Mahalanobis distance to the average of Europeans $< 6\sigma$) were excluded from further analysis. Finally, 1,341 subjects considered to be imaging outliers were excluded from the analysis. These were subjects for which average

head motion parameters, signal-to-noise ratio or discrepancy between T1-weighted and fMRI scan deviated from the median more than five times their median absolute variation.[3] These genotyping data were collected with two different array types - UK Biobank Axiom (UKBA) and the UK BiLEVE (UKBB) arrays - which cover 812,428 unique genetic markers and overlap 95% in SNP content.

*Subnetwork Definition*

The definition of resting-state networks (RSNs) based on the Yeo-Krienen 7-network atlas was carried out as described before.[4] Briefly, the Yeo-Krienen atlas contains a parcellation map of 7 large-scale functional resting-state networks, including the visual, somatomotor, dorsal attention, ventral attention, limbic, frontoparietal, and default mode network. An annotation file of the 7 functional networks was included for the fsaverage subject in the FreeSurfer Software package. For this, the surface-based annotation was translated to a 3D brain volume in volumetric space, in which each grey matter voxel was assigned a network label. Next, for each region in the Desikan-Killiany atlas, the ratio of voxels that belonged to each of the 7 RSNs was computed.[5] Using a majority vote approach, the label of the functional network corresponding to the majority of voxels was then assigned to that region.

All edges connecting areas belonging to the same network were assigned to it. The following regions in the aseg atlas were assigned to the subcortical network (all bilateral): Thalamus, Caudate, Putamen, Pallidum, Hippocampus, Amygdala, Accumbens-area.[6] Edges connecting cortical and subcortical edges were assigned to the cortical-subcortical category and the remaining to the between-network category. In total, the edges were divided into 10 different disjoint sets. The comprehensive list of subnetwork-edge pairings can be found in Supplementary Data 1.

*Subnetwork Enrichment Analysis*

We performed enrichment analysis to identify whether any particular subset of edges in

each RSN presents a statistically significant difference in SNP-heritability. The heritability estimation for each edge was divided by its standard deviation to create a Z-score. These scores were not independent of each other because (i) edges in the same RSN are more similar than those outside of the RSN; (ii) the data has strong spatial dependencies. Therefore, not accounting for these spatial dependencies (e.g. two-sample t-tests or simple linear regressions) would lead to a highly inflated type I error rate.

For each RSN, we used a linear regression $Z = \beta_0 + \beta_s X_s + \beta_r X_r + \epsilon_r$ on the n=3,321 edges as data points, with $\boldsymbol{Z}$ the vector of Z-scores for each edge, $X_s$ the average edge strength, and $\boldsymbol{X_r}$ a binary indicator variable scored 1 if the edge belonged to that RSN and 0 otherwise. The residuals were modelled as $\epsilon_r \sim MVN(0, {\sigma_r}^2 S)$, with ${\sigma_r}^2$ the residual variance parameter. The fixed $n$ x $n$ correlation matrix $\boldsymbol{S}$ was included to account for the dependencies between the heritability estimates of the edges and was set to the element-wise square of the phenotypic correlation matrix of the edges in the discovery sample.

The models were fitted using Generalized Least Squares. The parameter for each RSN represents the mean difference in strength of genetic signal (as quantified by their Z-score) of edges in that RSN compared to the other edges, after correcting for average edge strength, with positive corresponding to an enrichment of genetic signal in the RSN. Consequently, we performed a one-sided test of $H_0\colon \beta_r = 0$ against the alternative hypothesis $H_1\colon \beta_r > 0$, using a t-test with 2290 degrees of freedom (derived from the rank of $S$).

*Gene effect extent*

The effect extent for each gene was computed with the same methods as individual loci (Methods).

Briefly, SNP-wise mean model in MAGMA was run for all edges of the connectome to test the joint association of SNPs within each gene with the studied traits, using a reference panel derived from 10,000 unrelated UK Biobank participants.[7] This

analysis covered 18,850 protein-coding genes. For each gene, association results were compared across all 3,321 genome-wide association studies (GWGAS). To account for multiple testing, Benjamini–Hochberg false discovery rate (FDR) correction was applied across all 3,321 functional connectivity traits. The number of traits for which association with a gene remained significant after FDR correction was defined as its effect extent.

*LD-aware and LD-agnostic null models*

To evaluate whether the observed gene-set effect extents exceeded those expected by chance, we constructed three null models: LD-aware random gene-sets (all genes), LD-aware random gene-sets (brain-expressed genes only) and a LD-agnostic random gene-sets (brain-expressed genes only).

First, to account for linkage disequilibrium (LD) between genes, we estimated a gene–gene correlation matrix by running MAGMA on functional connectivity phenotypes. Initial columns from the resulting .raw MAGMA output were removed, and the truncated correlation matrix was converted into a full gene-gene correlation matrix representing LD relationships among genes ($\Sigma$).

Then, for each disorder, 10,000 random gene-sets of size $N$ (equal to the number of GWS genes for that disorder) were generated by sampling from a multivariate normal distribution with covariance structure defined by $\Sigma$. This ensured that random gene-sets reflected the observed LD structure. To further refine the null model, we restricted the sampling to 6,401 brain-expressed genes, as defined by SynGO[8], using the same LD-aware sampling procedure. Main results refer to benchmarking against a LD-aware set of random brain-expressed genes.

As a simpler background model for comparison, we generated LD-agnostic null sets by sampling $N$ random genes (without accounting for LD) from the SynGO background of highly brain-expressed genes.

*Null distribution generation procedure*

For both LD-aware models, random vectors $Z \sim MVN(0,\Sigma)$ were sampled for all genes, with $\Sigma$ being the gene-gene correlation matrix. This was done separately for all genes (18,623 in total) and for brain-expressed genes only (6,401 genes). Each element of the sampled vector corresponds to one gene. The values in $Z$ were ranked, and genes were ordered accordingly. To construct a random gene-set of size $N$ (equal to the number of genome-wide significant genes for the given phenotype), we selected the first $N$ genes. As the probability of extraction is random other than for LD (through $\Sigma$), this ranking-based selection preserves the LD structure while selecting random genes.

The same sampling and selection procedure was applied repeatedly to generate 10,000 random gene-sets per phenotype for each null model: LD-aware (all genes), LD-aware (brain-expressed genes only) and LD-agnostic (brain-expressed genes only, used here for comparison). These iterations were used to calculate empirical null distributions for the expected mean effect extent of random gene-sets under each model.

**Supplementary Note 1 - Sample Demographics**

After quality control, a total of 28,159 subjects of European ancestry were considered for this study. These comprised a discovery and replication sample of 24,451 and 3,708 unique unrelated individuals, respectively. A complete description of the sample demographic characteristics can be found in Supplementary Note Table 1.1. Field codes for all variables used in this study can be found on Supplementary Note Table 1.2. Age, head motion and intracranial volume did not follow a normal distribution (Supplementary Note Figure 1.1). Association testing showed a small significant difference between groups for age. The standardised mean difference for age between discovery and replication groups was 0.182, which was considered a small effect size and unlikely to confound the genetic analyses.

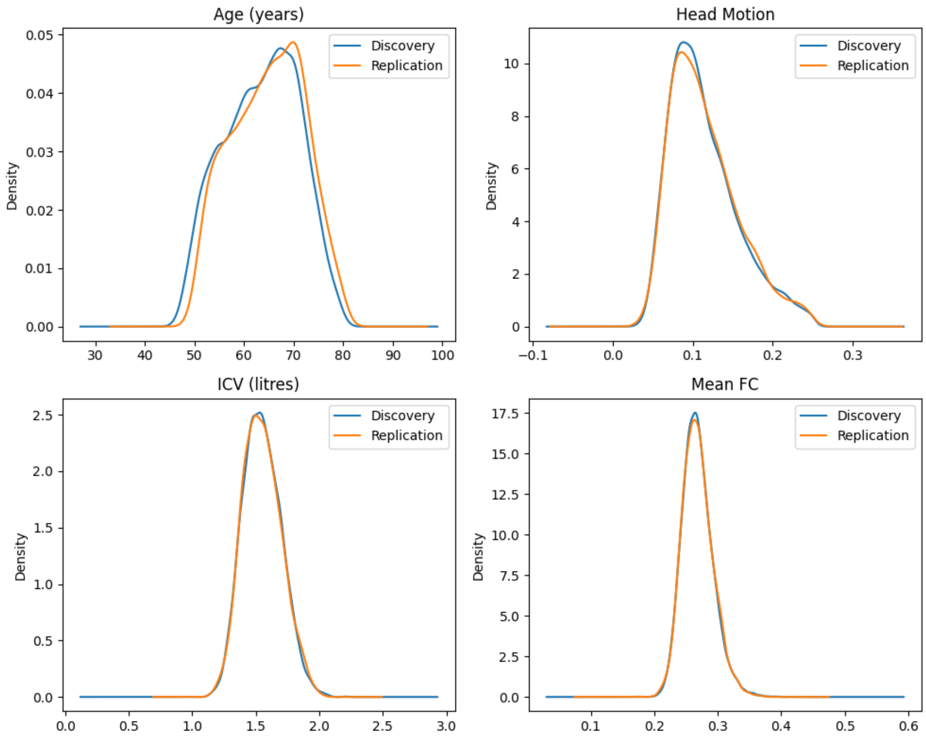


**Supplementary Note Figure 1.1. Density plots for non-normal demographic variables.**

Age (top left), Head Motion (top right), Mean of Non-Negative functional connections (bottom left) and Intracranial Volume (bottom right). The density curves are plotted in blue for the discovery and orange for the replication samples.

**Supplementary Note Table 1.1. Description of the demographic sample and significance of association testing.**

Chromosomal sex, handedness and array type are categorical variables, association was tested using the chi-squared test. The Kruskal-Wallis H test calculates *p*-values for age, head motion and intracranial volume sample differences. Reported *p*-values are Bonferroni-adjusted for the number of tests. Q1: first quartile; Q3: third quartile; UKBA: UK Biobank Axiom; UKBB: UK BiLEVE.

| | | Overall | Discovery | Replication | P-Value (adjusted) |
|---|---|---|---|---|---|
| **N** | | 28159 | 24451 | 3708 | |
| **Chromosomal Sex, N (%)** | Female | 15197 (54.0) | 13151 (53.8) | 2046 (55.2) | 0.818 |
| | Male | 12962 (46.0) | 11300 (46.2) | 1662 (44.8) | |
| **Age, median [Q1,Q3]** | | 64.0 [58.0,70.0] | 64.0 [58.0,69.0] | 65.0 [59.0,71.0] | $4.1 \times 10^{-22}$ |
| **Head Motion, median [Q1,Q3]** | | 0.1 [0.1,0.1] | 0.1 [0.1,0.1] | 0.1 [0.1,0.1] | 1.000 |
| **Handedness, N (%)** | Left | 2653 (9.4) | 2269 (9.3) | 384 (10.4) | 0.276 |
| | Non-left | 25506 (90.6) | 22182 (90.7) | 3324 (89.6) | |
| **Array Type, N (%)** | UKBA | 25653 (91.1) | 22273 (91.1) | 3380 (91.2) | 1.000 |
| | UKBB | 2506 (8.9) | 2178 (8.9) | 328 (8.8) | |
| **Intracranial Volume (litres), median [Q1,Q3]** | | 1.5 [1.4,1.7] | 1.5 [1.4,1.7] | 1.5 [1.4,1.7] | 1.000 |
| **Mean Functional Connectivity, median [Q1,Q3]** | | 0.3 [0.3,0.3] | 0.3 [0.3,0.3] | 0.3 [0.3,0.3] | 1.000 |

**Supplementary Note Table 1.2. UKB field codes for covariates.**

| Field | Description |
|---|---|
| f.31.0.0 | Self-reported sex |
| f.54.2.0 | Screening and Imaging Centre |
| f.21003.2.0 | Age at imaging visit |
| f.1707.0.0 | Handedness |
| f.22000.0.0 | Genomic batch |
| f.25744.2.0 | Signal-to-Noise Ratio |
| f.25756.2.0 | Position of the table in the X axis |
| f.25757.2.0 | Position of the table in the Y axis |
| f.25758.2.0 | Position of the table in the Z axis |
| f.25759.2.0 | Position of the coil relative to table |
| f.25923.2.0 | Time-to-Echo |
| f.25929.2.0 | Intensity Scaling |
| f.26521.2.0 | Intracranial Volume |

**Supplementary Note 2 - Subnetwork Enrichment Analyses**

In addition to analyses on subnetwork enrichment (Results; Sup. Tab. 1), enrichment in intrahemispheric, interhemispheric, subcortical and cortico-subcortical edges (referred to as edge topology; Supplementary Note Table. 2.1) was also calculated. None of the enrichment values was significant (Supplementary Note Figure. 2.1).

**Supplementary Note Table 2.1. Enrichment for different topologies of edges.** LDSC SNP-heritability estimate of the edges in each topology category (mean ± standard deviation). $N_{edges}$ is the number of edges in each subnetwork. Enrichment is the *t*-statistic of the subnetwork enrichment test (Supplementary Methods). *P*-values are unadjusted, no result is nominally significant.

| Topology | $h^2_{SNP}$ | $N_{edges}$ | Enrichment $t(269)$ | P-value |
|---|---|---|---|---|
| Interhemispheric | 0.042 ± 0.029 | 1156 | 0.497 | .310 |
| Left | 0.044 ± 0.029 | 561 | 0.275 | .392 |
| Right | 0.043 ± 0.030 | 561 | -0.675 | .641 |
| Subcortical | 0.019 ± 0.019 | 91 | -0.820 | .794 |

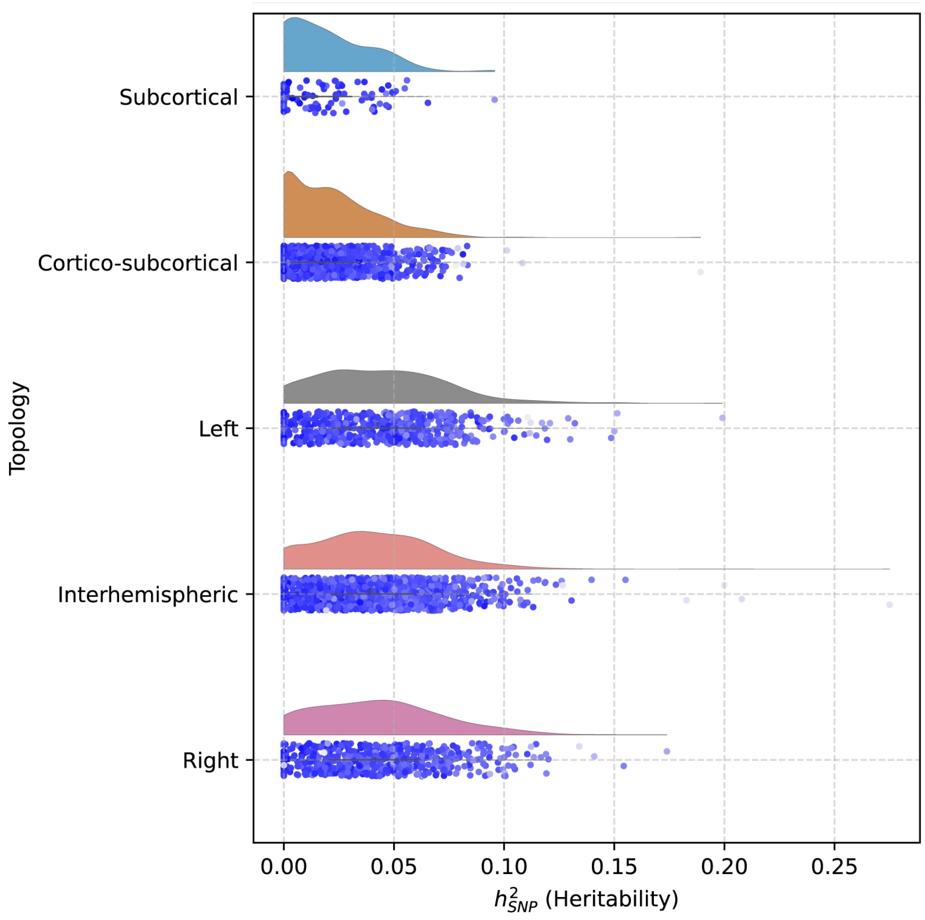


**Supplementary Note Figure 2.1. Raincloud plots for heritability estimates grouped per topology.**

Half-violin plots represent the distribution of edge LDSC SNP-heritability. Each point below the violin plot is the heritability estimate for each edge belonging to the network. The shade of points in the jitter plot is the standard deviation of the estimation: lighter - higher uncertainty.

**Supplementary Note 3 - Reliability Analysis**

*Relationship between heritability and measurement reliability*

The relationship between test–retest reliability of edges and heritability was investigated to characterise the power in this study. From our total sample of 28,159 individuals, 3,018 had a second scan available with similar preprocessing. The intraclass correlation coefficient $\mathrm{ICC}(3,1)$ was calculated for each of the 3,321 edges and used to access univariate test-retest reliability.

The ICC values ranged from 0 to 0.60 with a median of 0.19. When only considering heritable edges, the median $\mathrm{ICC}(3,1)$ is 0.29 (range = [0.04; 0.60]). When only considering edges for which SWS associations were found, the median ICC is 0.41 (range = [0.20; 0.60]). Spearman's correlation between the z-values of the LDSC SNP-heritability and ICC values were calculated to be 0.68, indicating a high correlation between the certainty of the SNP-heritability estimation and the reliability of the phenotypic measurement (Supplementary Note Figure 3.1). These results showed heritability estimates are higher for those edges with the highest measurement reliability, meaning that noisy measurements tend to yield lower heritability estimates reflecting a relative loss of power. Despite the substantial correlation between these two sets of values, measurement reliability is not the only factor influencing the estimation of SNP-heritability, as different edges of the connectome may be affected by genes and the environment to different extents.

*Global Signal Regression Effect*

To explore the potential impact of GSR in the genetic signal of FC, we have repeated a representative set of GWAS analysis without GSR. First, we identified the connections most affected by its application (Supplementary Note Figure 3.2 shows the correlation between connectivity with and without GSR in the UKB sample). From all 1,083 significantly heritable edges in this study, we selected a representative set: the edge with the minimum and maximum change with versus without GSR, and one edge per decile

of change (Supplementary Note Table 3.1). We then repeated 11 GWAS for these edges and evaluated the effect of GSR by calculating genetic correlations.

Out of the 11 GWAS investigated, 10 showed a lower SNP-heritability estimate when GSR was not applied. This is in line with the analysis above showing that edges with a higher test-retest reliability have higher SNP-heritability estimates. As GSR may mitigate physiological and motion-related noise, its application improves the signal-to-noise ratio by removing transient (i.e. session/state-specific) noise and, therefore, is suitable for genetic analyses. However, in those traits which retained enough heritability to perform genetic correlations, this is very high ($r_g \sim 1$), indicating that the genetic signal we detect is similar in both analyses.

**Supplementary Note Table 3.1. Comparison of SNP-heritability estimates after the application of GSR.**

Decile: Decile of change of measure with and without GSR (e.g., 10% correspond to an edge in the bottom 10% change after GSR, Min: most similar edge, Max: most affected edge). Corresponding Edge: edge of connection. Mean Difference: Mean difference of edge strength across all subjects before and after applying GSR. $h^2_{SNP}$ before GSR: SNP-heritability value before applying GSR. $h^2_{SNP}$ after GSR: SNP-heritability value after applying GSR. Genetic Correlation: LDSC genetic correlation estimate of GWAS before and after applying GSR.

† LDSC Genetic correlation and heritability are unbounded estimators. Therefore, heritability might be lower than 0 and genetic correlations higher than 1, when power is low. Correlations estimations were capped to 1.00 and heritability at 0.

| Decile | Corresponding Edge | Mean Difference | Phenotypic Correlation | $h^2_{SNP}$ before GSR | $h^2_{SNP}$ after GSR | Genetic Correlation |
|---|---|---|---|---|---|---|
| Min | ctx-lh-frontalpole<br>ctx-lh-precuneus | -0.025 | 0.74 | 0.045 | 0.012 | 1.00† |

| | | | | | | |
|---|---|---|---|---|---|---|
| 10% | ctx-rh-superiortemporal<br>ctx-lh-entorhinal | 0.075 | 0.77 | 0.049 | 0.039 | 1.00† |
| 20% | ctx-rh-rostralanteriorcingulate<br>ctx-lh-middletemporal | 0.11 | 0.77 | 0.046 | 0.00† | NA |
| 30% | ctx-rh-rostralanteriorcingulate<br>ctx-lh-caudalmiddlefrontal | 0.15 | 0.75 | 0.046 | 0.022 | 1.00† |
| 40% | ctx-rh-postcentral<br>Left-Caudate | 0.19 | 0.77 | 0.058 | 0.00† | NA |
| 50% | ctx-rh-paracentral<br>ctx-lh-posteriorcingulate | 0.22 | 0.75 | 0.047 | 0.00† | NA |
| 60% | ctx-rh-parstriangularis<br>ctx-lh-precentral | 0.26 | 0.75 | 0.066 | 0.00† | NA |
| 70% | ctx-rh-rostralmiddlefrontal<br>ctx-lh-isthmuscingulate | 0.29 | 0.79 | 0.08 | 0.058 | 0.97 |
| 80% | ctx-rh-inferiortemporal<br>ctx-lh-parsopercularis | 0.33 | 0.68 | 0.034 | 0.019 | 1.00† |
| 90% | ctx-rh-superiorparietal<br>ctx-rh-parsorbitalis | 0.39 | 0.63 | 0.044 | 0.017 | 1.00† |
| Max | ctx-rh-middletemporal<br>ctx-lh-superiorparietal | 0.63 | 0.62 | 0.066 | 0.067 | 1.00† |

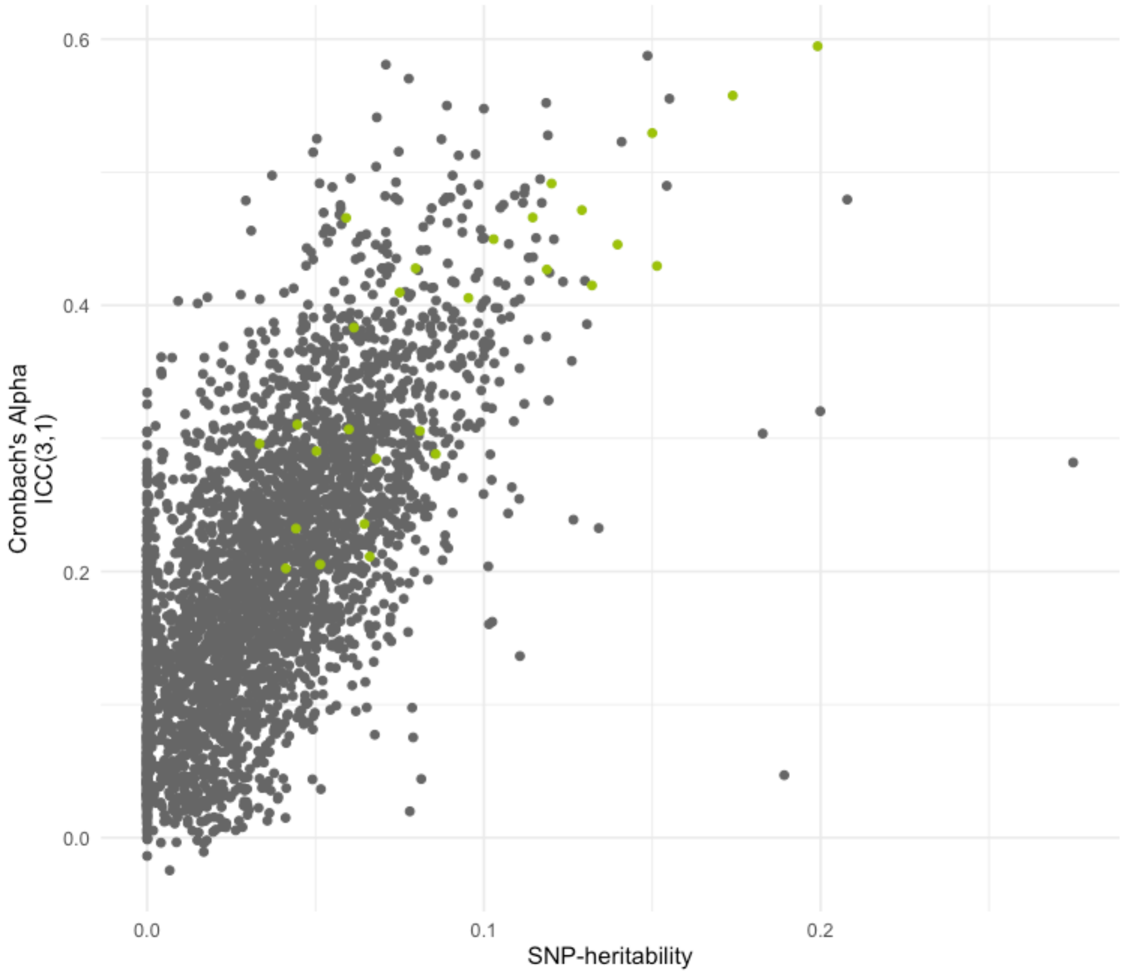


**Supplementary Note Figure 3.1. Relationship between measurement reliability and SNP-heritability.**

Comparison of ICC(3,1) with SNP-heritability estimation. Note that the heritability estimates are variable, reflecting a possibly different contribution of common variants to different brain connections, even among those for which the measurement is the most reliable.

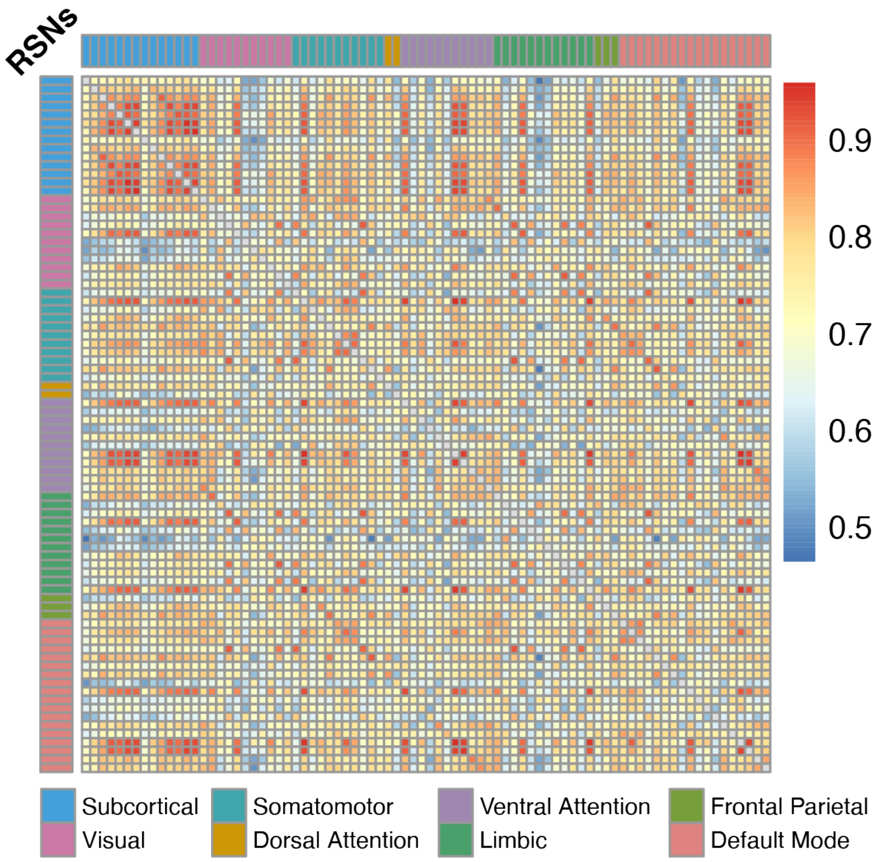


**Supplementary Note Figure 3.2. Global signal regression (GSR) effect comparison.** Each element *i,j* of the matrix provides the Pearson correlation between the strength of the edge connecting area *i* and *j* before and after applying GSR. The colours show the resting state networks (RSNs) to which row *i* and column *j* belong.

**Supplementary Note 4 - Validation of overlapping loci**

SNP prioritisation and loci co-localisation were carried out to ensure the overlapping loci across GWAS have the same source of genetic signal. The determination of common likely causal SNPs for different locus-edge associations was performed by colocalising overlapping loci. For this purpose, two loci from two different GWAS were considered to overlap if their respective lead SNPs were separated by less than 250K base pairs. If a locus was small (<250K base pairs) a 250K region around the centre of the locus was considered (**Supplementary Note Figure. 4.1**). A total of 79 replicated locus-edge were found to be associated with three overlapping loci: 11 with *PAX8* (*2:113963070:114213070*), 13 with *EphA3* (*3:89451721:90010903*) and 3 with *THBS1* (*15:39514832:39764832*). Summary statistics in the overlapping loci were filtered to contain only SNPs that reach the marginal significance threshold of $\alpha = 0.05$ in the three loci of interest. Estimation of the LD reference panel consisting of all the individuals in each discovery GWAS was carried out on LDStore2 (v2.0).[9] The resulting LD reference panel was used to fine-map each of the overlapping loci with FINEMAP (v1.4.1), using a threshold of a maximum of 10 causal variants per locus.[9] Furthermore, the probability of the overlapping loci having the same causal variant across GWAS was estimated using approximate Bayes factor co-localisation, implemented in coloc (v5.2.2).[10] Two locus-trait associations were considered colocalised if these had a 90% probability of sharing a single causal variant ($H_4 > 0.9$).

For the three overlapping loci, all 78 locus-trait associations were predicted to have only one causal variant. For the *PAX8* and *THBS1* loci, all edges were found to be co-localised ($H_4 > 0.97$ for all comparisons). For the EphA3 locus, 85% of the 11 edges co-localised (66 out of 78 unique edge pairs). The associations that did not co-localise were always with the intrahemispheric connectivity between the right supramarginal and medial temporal gyri (**Supplementary Note Figure. 4.2**). All of these had moderate (> 45%) $H_4$ probabilities. In case $H_4 < 0.9$, $H_3$ would be the second highest probability by several orders of magnitude. These all happened for the same edge, indicating that this

edge is likely associated with a different SNP in this locus (**Supplementary Note Figure. 4.3**). However, FLAMES mapped the same gene to this locus, showing that downstream effects are likely to be converging on the same gene. Taken together, the results reflected a high probability that the genetic effects we observed in the 22 individual edge-GWAS were all associated with the same genetic variants within the three overlapping loci. This brings further evidence for the pleiotropy of these discovered loci for several functional links across the human brain.

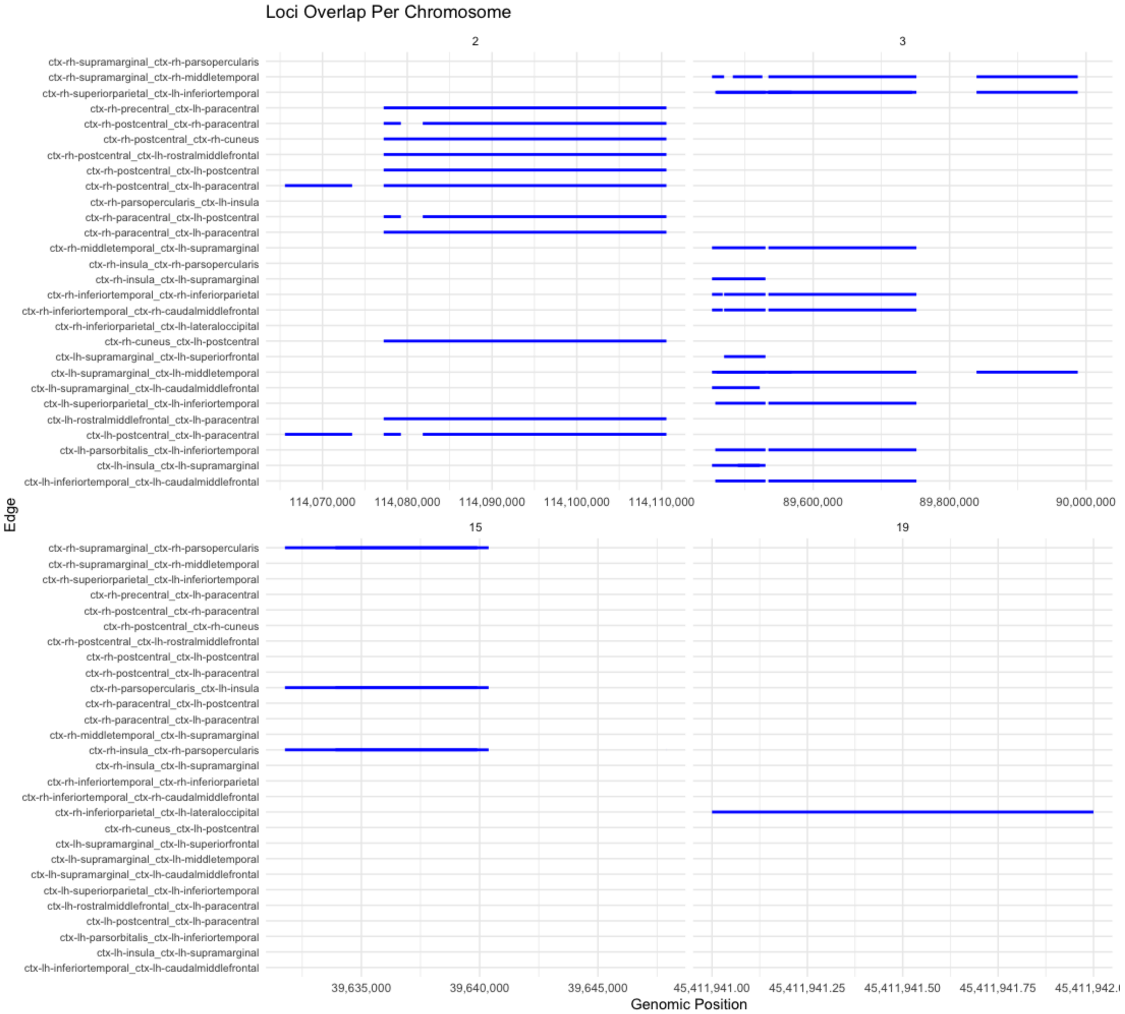


**Supplementary Note Figure 4.1. Visualisation of locus boundary overlap.**

Each blue line represents one significant replicated locus association with the respective edge. Locus edge associations are found in close proximity across the four loci, being mapped into one overlapping locus encompassing the different locus-edge associations.

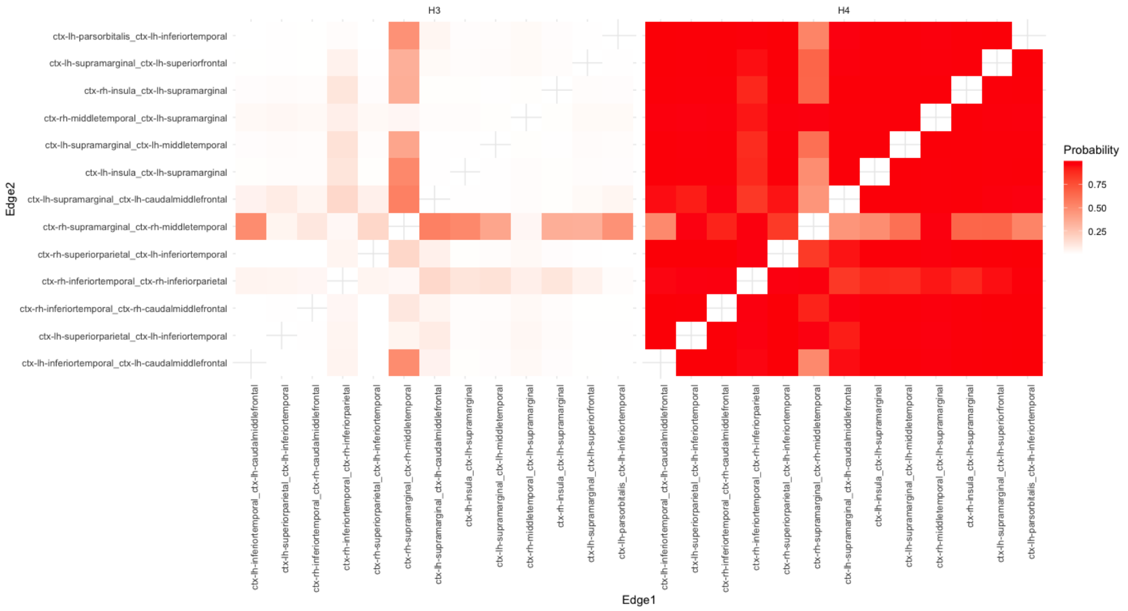


**Supplementary Note Figure 4.2. Heatmap for $H_3$ (left) and $H_4$ (right) probability in coloc for all unique pairs of edges associated with the EphA3 locus (*3:89451721:90010903*).**

All pairs of edges are found to have a high probability of having the same putative causal variant, except for the right supramarginal and medial temporal gyri (right). This edge is found to have a modest probability of having a unique causal variant in this locus (left).

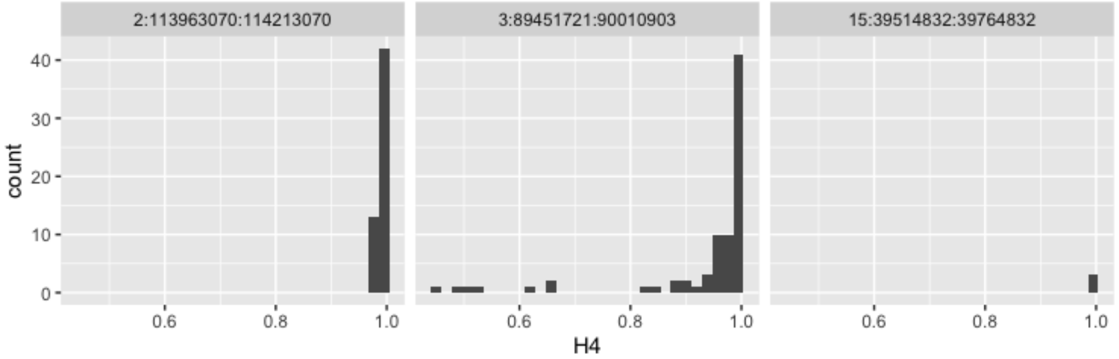


**Supplementary Note Figure 4.3. Histograms for $H_4$ probability in coloc for all unique pairs of edges associated with the *PAX8* (*2:113963070:114213070*), *EphA3* (*3:89451721:90010903*) and *THBS1* locus (*15:39514832:39764832*).**

Locus positions according to GRCh37 build.

**Supplementary Note 5 - Comparison of phenotypic definitions**

*Comparison with edge-wise ICA-derived functional connectivity*

All GWS findings of this study were compared against Zhao et al (2022)[11] to assess the impact of the choice of a parcellation-based versus an ICA-based edge definition. We compiled all genome-wide significant (GWS; $p < 5\times10^{-8}$) associations with edge-wise functional connectivity reported by Zhao et al., totalling 232 loci with unique lead SNPs. These were compared against the 887 unique GWS loci identified in our study. Overlap between studies as loci with boundaries intersecting within a 250 kb window, allowing for variation in LD structure.

This comparison revealed substantial concordance between our findings and Zhao et al (Supplementary Note Figure 5.1). Out of all GWS loci in our study, 33% were also reported by Zhao et al. Among the four loci reaching study-wide significance (SWS; $p < 1.5 \times 10^{-11}$) in our study, three were also SWS in Zhao et al., and the fourth was reported at GWS. Conversely, 71% of Zhao et al. GWS loci were replicated at GWS in our dataset. In addition, we identified novel 594 GWS loci not previously reported for edge-wise connectivity by Zhao et al. Taken together, these results suggest that, while ICA and parcellation-based approaches capture different facets of brain connectivity, our parcellation-based analysis shows that increased resolution can replicate established findings while also revealing novel associations.

*Comparison with average resting-state network functional connectivity*

To analyse the impact of adopting a less granular phenotype definition, we ran LDSC genetic correlations between each edge-GWAS and GWAS of functional resting-state networks (RSNs) from Tissink et al. (2023).[12] Given the harmonised preprocessing across both studies, comparing the genetic architectures of RSN with the structures they integrate allows to contrast different granularities of phenotypic definitions.

The *p*-values of the genetic correlation estimates were FDR-corrected, and the significance threshold was set at 0.05/7 to account for the seven RSNs tested. Out of all

tests performed, we identified 11 significant genetic correlations with the somatomotor network, 20 with the default mode network (DMN), and 4 with the limbic network. All significant correlations with the somatomotor network involved edges within the network. Interestingly, the strongest correlations were between the bilateral pre-, para- and postcentral gyri, implying that global somatomotor signal mostly captures primary sensorimotor function. For the DMN, 6 of the 20 significant correlations were with edges belonging to the DMN, while the remaining 14 involved edges between networks. In the limbic network, 2 of the 4 significant correlations were with edges within the network, and the other 2 with edges between networks. Additionally, for the ventral attention network, a number of edges showed subthreshold correlations that suggest a similar pattern, indicating that some network-specific correlations may not reach significance due to limited power or lower heritability of individual edges or of the respective RSN (see Supplementary Note Figure 5.2 for a summary of the results). All significant or suggestive within-network associations were positively correlated with the respective RSN, which follows the expected direction. Overall, these findings indicate there is convergence and difference of edge-level and network-level genetic architecture.

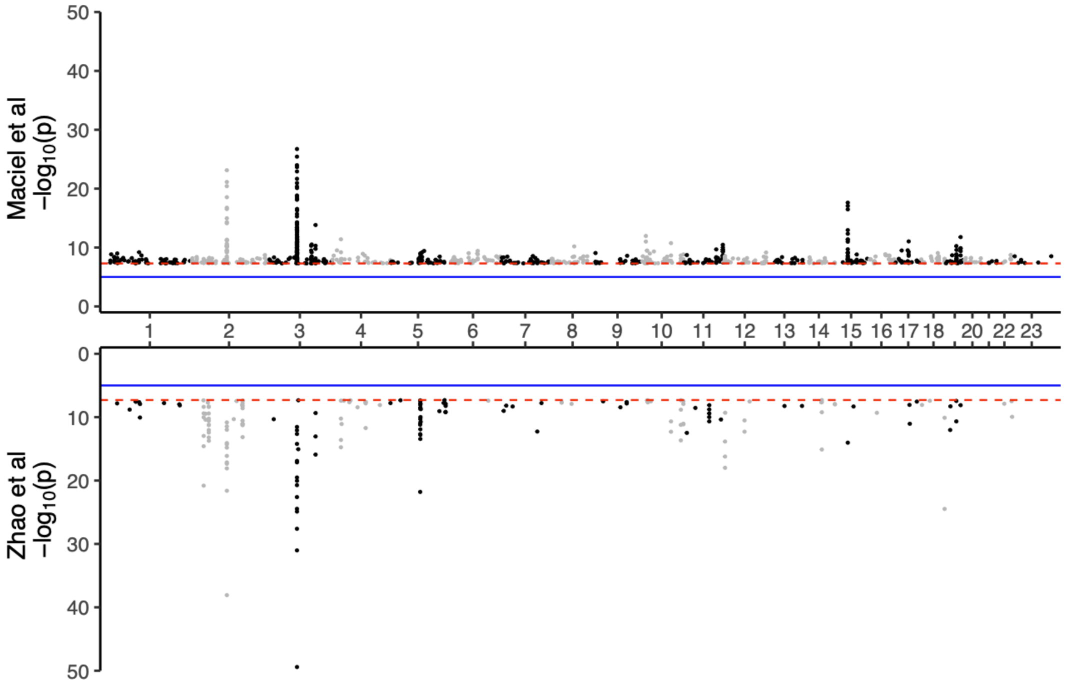

**Supplementary Note Figure 5.1. Comparison of GWS loci with ICA-based phenotype definition.**

Miami plot of all lead SNPs reported in the present manuscript (top) and in Zhao et al.[11] (bottom). Only the lead SNP are shown. In case the same lead SNP was reported for two or more traits, the lowest p-value is represented.

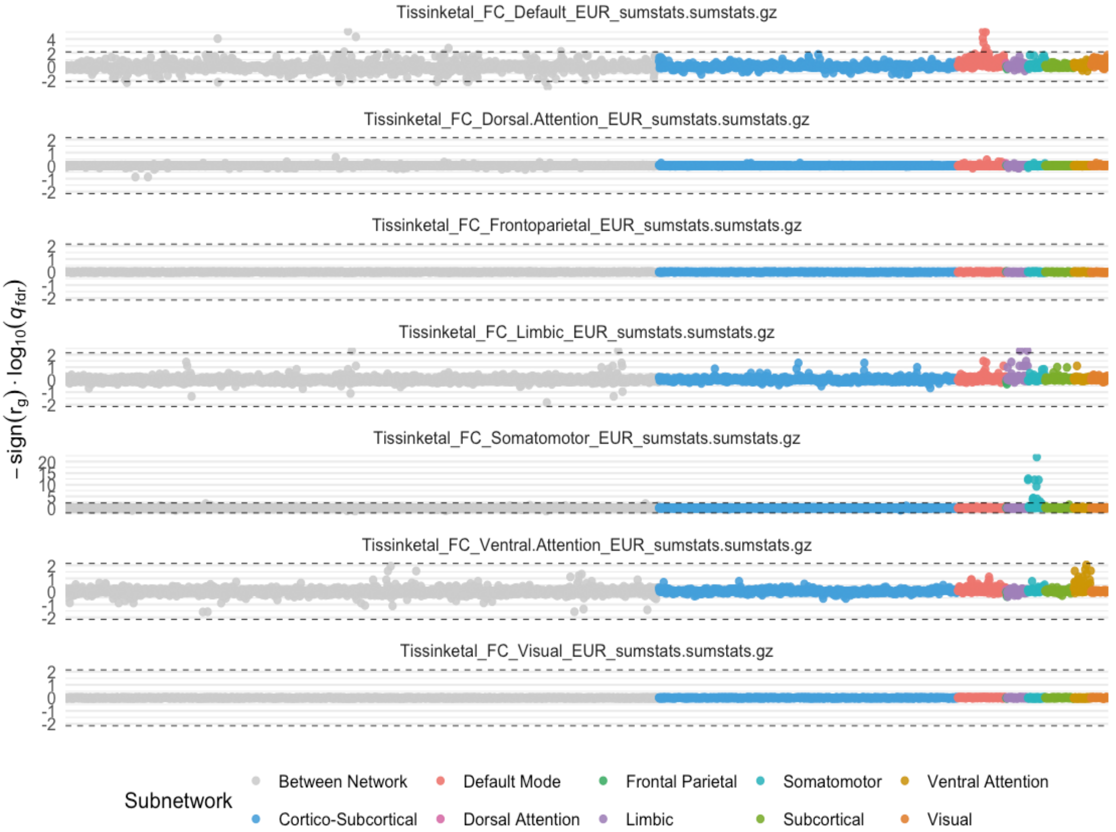


**Supplementary Note Figure 5.2. Similarity of edge and RSN genetic architecture.**

Comparison performed with summary statistics from Tissink et al.[12] LDSC genetic correlations between GWAS were performed on FC of RSN and in each of 3,321 edges included in this study. All q-values presented in the figure correspond to the Benjamini-Hochberg FDR-corrected p-values of the LDSC genetic correlation estimate.

## Supplementary Note 6 - Genetic overlap analyses of functional connectivity and disorder

*GWAS and Gene-sets for disorder*

All most recent case-control disorder GWAS per working group in the Psychiatric Genetic Consortium were considered for this study. Summary statistics were uploaded to FUMA, where MAGMA was used to run GWGAS for all protein-encoding genes with a window of 0Kb around the gene and using the 1KG EUR reference panel as an LD reference.[2,7,13] GWGAS with less than 5 significant genes were not kept for downstream analyses because of the small size of the gene-set. After this filtering, total of 8 different datasets were kept: Attention Deficit/Hyperactivity Disorder (ADHD; **Supplementary Note Figure. 6.1**),[14] Anorexia (**Supplementary Note Figure. 6.2**),[15] Alzheimer's Disorder (AD; **Supplementary Note Figure. 6.3**),[16] Bipolar Disorder (BIP; **Supplementary Note Figure. 6.4**),[17] Depression (Dep; **Supplementary Note Figure. 6.5**),[18] Substance Use Disorder (SUD; **Supplementary Note Figure. 6.6**)[19] and Schizophrenia (**Supplementary Note Figure. 6.7**).[20] A gene-set was built for each disorder by joining all the genes with a p-value lower than 0.05/18,623 (corrected for number of genes tested).

*LDSC analysis*

LDSC genetic correlations were computed between all edges and the 7 neuropsychiatric traits used. Results are summarised in **Suppl. Fig. 7** and **Suppl. Table 6**. Only schizophrenia showed FDR-significant genetic correlations with 21 edges (**Supplementary Note Figure 6.1**). All other traits presented at least one nominally significant correlation, but none survived multiple-testing correction. Similarly with the analysis performed in comment 4., we expect these correlations to become more significant with increased sample sizes of the FC GWAS.

*Mendelian Randomisation*

To investigate suggestive causality, Generalised Summary-data-based Mendelian Randomization was run with GSMR on all edge-GWAS which showed a nominally significant genetic correlation with a disorder-GWAS as calculated above.21 We note that a study with an experimental design is more appropriate to investigate causality, and MR only provides evidence of suggestive causality. A minimum of 5 independent GWS (i.e., $p < 5\times10^{-8}$) SNPs were considered for each analysis. Reverse analyses were only run if at least one significant locus was identified for the respective edge-GWAS. This accounted for 241 GSMR analyses: 6 for AD, 29 for ANO, 55 for BIP, 31 for MDD, 93 for SCZ and 27 for SUD. Both the forward (SNP → Edge → Disorder) as well as the reverse causality (SNP → Disorder → Edge) chains were tested.

No edge-disease pair had enough instruments post-filtering to perform the forward analysis. Twenty nominally significant reverse analyses were found: 1 for AD (positive beta), 9 for BIP (mixed sign), 4 for MDD (negative betas), 6 for SCZ (mixed sign) and 1 for SUD (positive beta). The most significant *p*-value ($p = 5\text{x}10^{-4}$) was found for a putative causal effect of MDD on the edges connecting the right fusiform gyrus and the right caudal medial frontal lobe. No test survived FDR correction across the successfully performed analyses. As the forward analysis could not be performed due to low locus yield, the results of the reverse analysis were not found to be sufficient to draw a conclusion about causality.

*Disorder gene-set effect extent comparison*

Permutation testing was performed to determine whether disorder genes are expected to affect more of the functional connectome than random genes (**Supplementary Methods**). On average, if a gene is known to be involved in disorder, it was found to be affecting more of the functional connectome for anorexia ($p = 0.03$: p-value for a random LD-aware gene-set of brain expressed genes), Alzheimer's disease ($p < 1\text{x}10^{-4}$), and schizophrenia ($p < 1\text{x}10^{-4}$). After correcting for multiple testing for the number of

disorders, the gene-sets for AD and schizophrenia were still significant for having a larger effect on the connectome than a LD-equivalent set of highly brain-expressed genes. Results remain nominally significant when considering all protein-coding genes in the genome. Results are summarised in **Sup. Fig. 5** and **Supplementary Note Table 6.1**. These results strengthen the relationship between genes, connectivity and disorder, showing that there is pervasive pleiotropy between neuropsychiatric disorders and the resting-state of the brain.

**Supplementary Note Table 6.1. Permutation testing results for the effect extent of different neuropsychiatric disorders.**

P-values for the different null models: LD-aware with a background of all protein-coding genes and LD-agnostic and LD-aware with a background of highly brain expressed genes. $N_{genes}$ is the number of significant genes per disorder.

| Source GWAS | $N_{Genes}$ | $p$-value LD-aware protein-coding | $p$-value LD-aware brain |
|---|---|---|---|
| ADHD (Demontis 2022) | 46 | .089 | .13 |
| Anorexia (Watson 2019) | 38 | .023 | .033* |
| AD (Wightman 2021) | 97 | >.0001** | .0001** |
| BIP (Mullins 2021) | 216 | .090 | .14 |
| Depression (Howard 2019) | 133 | .087 | .13 |
| SUD (Hatoum 2023) | 42 | .113 | .267 |
| Schizophrenia (Trubetskoy 2022) | 753 | 0.01* | >.0001** |

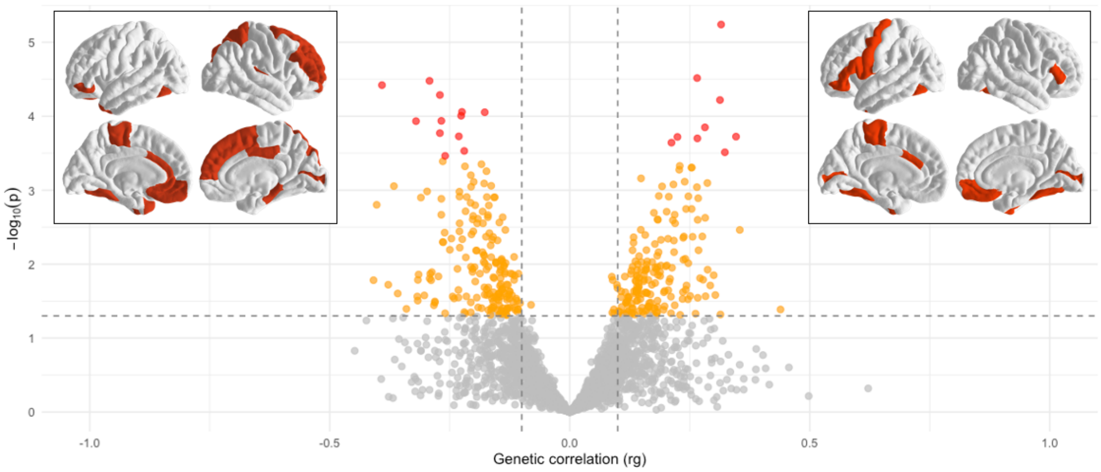


**Supplementary Note Figure 6.1. Significant genetic correlations between schizophrenia and FC edges.**

On the left and right are depicted the areas involved in the negative and positive genetic correlations with schizophrenia, respectively. Only FDR-significant edges (in red) are depicted.

**Supplementary Note Figure 6.2. ADHD GWGAS.**

Significant genes are annotated. Horizontal line denotes significance level 0.05/18623 = $2.65 \times 10^{-6}$.

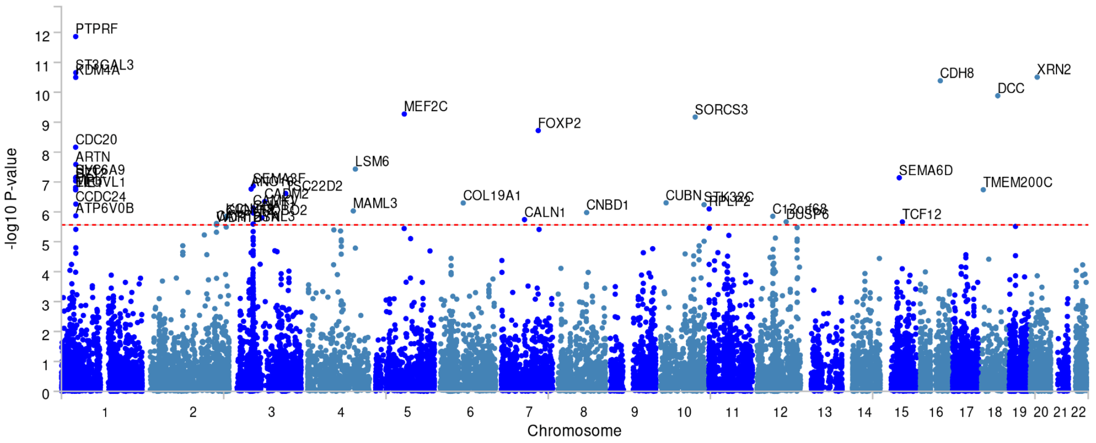

**Supplementary Note Figure 6.3. Anorexia GWGAS.**

Significant genes are annotated. Horizontal line denotes significance level 0.05/18623 = $2.65 \times 10^{-6}$.

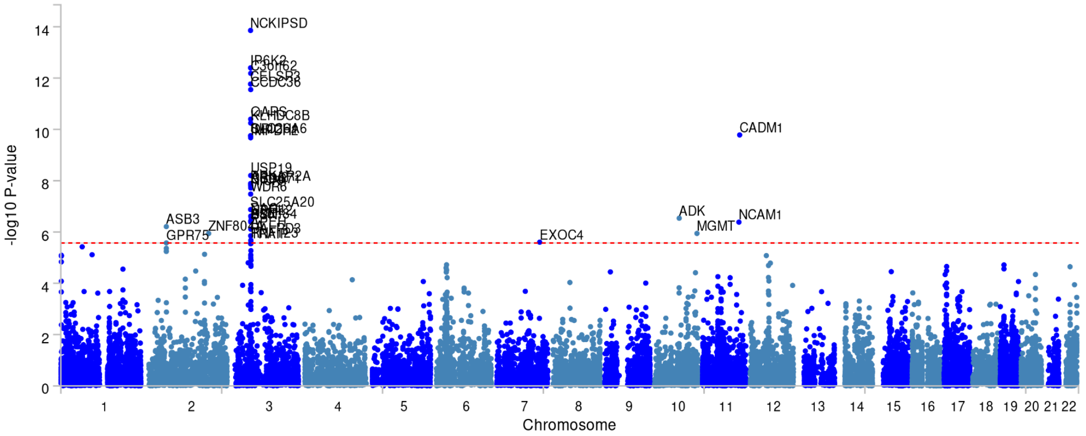


**Supplementary Note Figure 6.4. Alzheimer's Disorder GWGAS.**

Significant genes are annotated. Horizontal line denotes significance level 0.05/18623 = $2.65 \times 10^{-6}$.

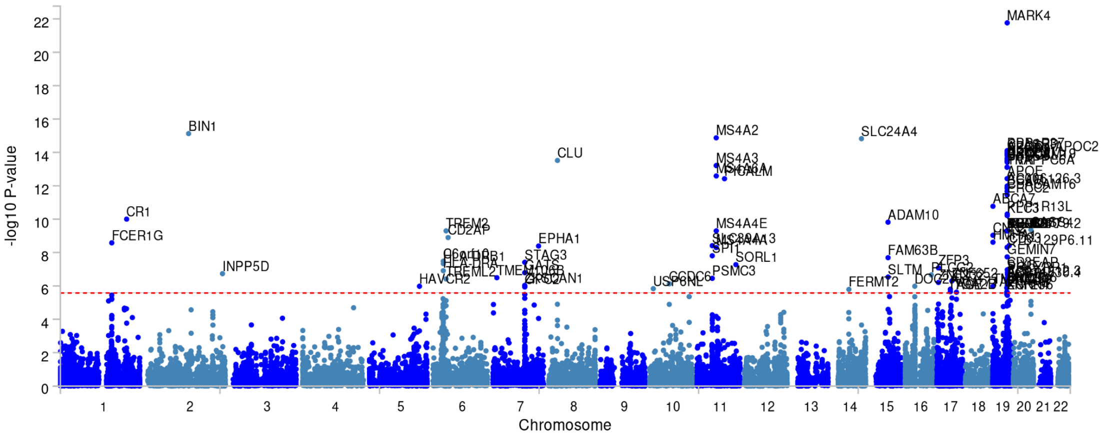


**Supplementary Note Figure 6.5. Bipolar Disorder GWGAS.**

Significant genes are annotated. Horizontal line denotes significance level 0.05/18623 = $2.65 \times 10^{-6}$.

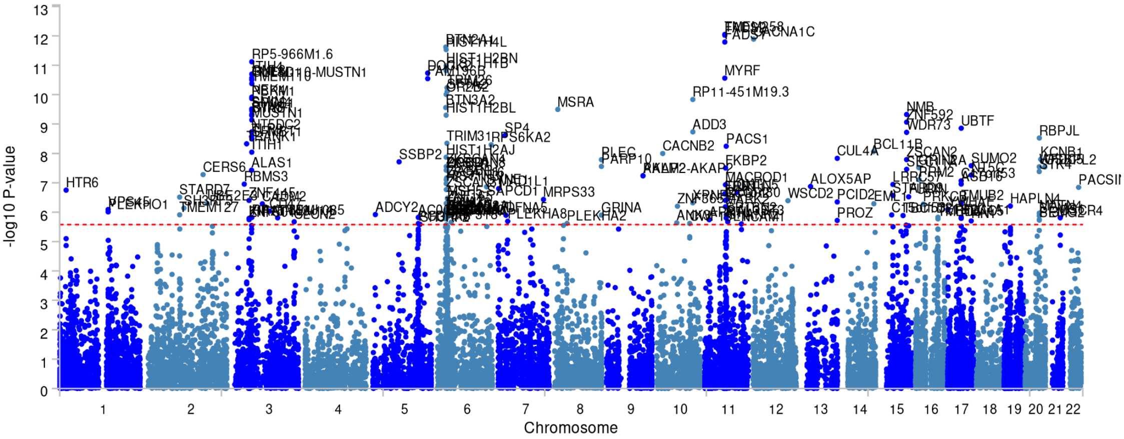


**Supplementary Note Figure 6.6. Depression GWGAS.**

Significant genes are annotated. Horizontal line denotes significance level 0.05/18623 = $2.65 \times 10^{-6}$.

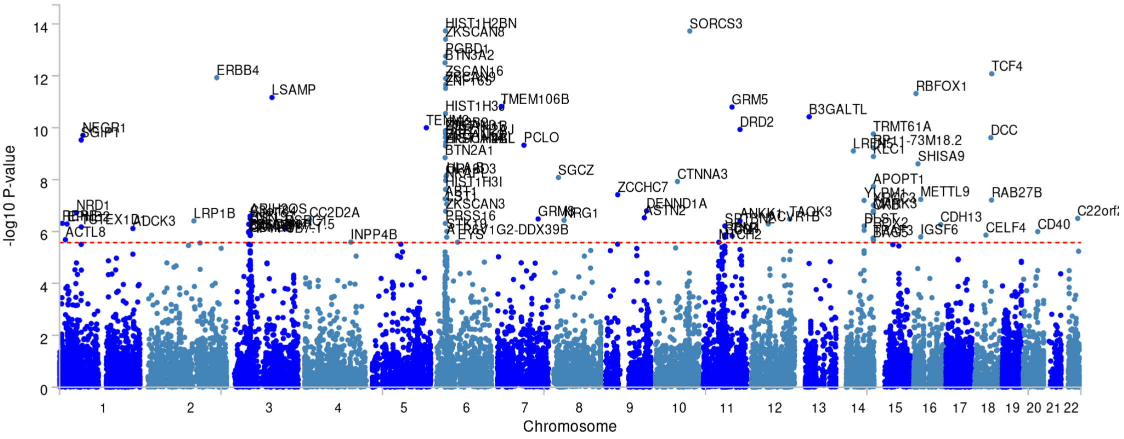


**Supplementary Note Figure 6.7. Substance Use Disorder GWGAS.**

Significant genes are annotated. Horizontal line denotes significance level 0.05/18623 = $2.65 \times 10^{-6}$.

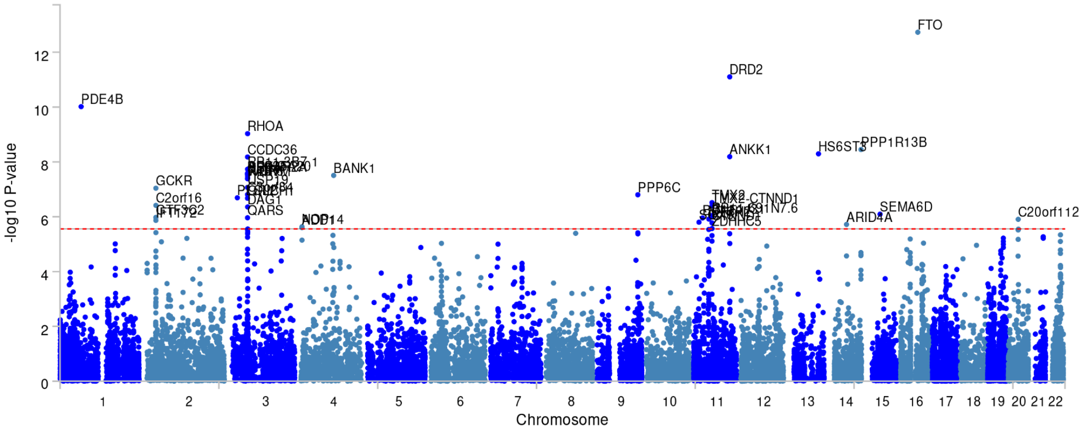


## Supplementary Note Figure 6.8. Schizophrenia GWGAS.

Top 100 Significant genes are annotated. Horizontal line denotes significance level 0.05/18623 = $2.65 \times 10^{-6}$.

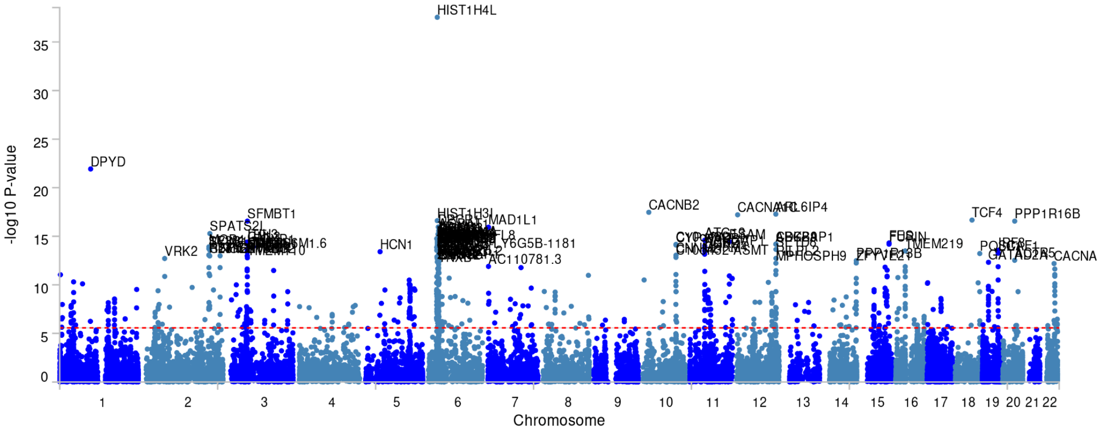

**References**

1. Abraham, G., Qiu, Y. & Inouye, M. FlashPCA2: principal component analysis of Biobank-scale genotype datasets. *Bioinformatics* **33**, 2776–2778 (2017).
2. Fairley, S., Lowy-Gallego, E., Perry, E. & Flicek, P. The International Genome Sample Resource (IGSR) collection of open human genomic variation resources. *Nucleic Acids Res.* **48**, D941–D947 (2020).
3. Alfaro-Almagro, F. *et al.* Confound modelling in UK Biobank brain imaging. *NeuroImage* **224**, 117002 (2021).
4. Wei, Y. *et al.* Genetic mapping and evolutionary analysis of human-expanded cognitive networks. *Nat. Commun.* **10**, 4839 (2019).
5. Desikan, R. S. *et al.* An automated labeling system for subdividing the human cerebral cortex on MRI scans into gyral based regions of interest. *NeuroImage* **31**, 968–980 (2006).
6. Fischl, B. *et al.* Whole Brain Segmentation: Automated Labeling of Neuroanatomical Structures in the Human Brain. *Neuron* **33**, 341–355 (2002).
7. de Leeuw, C. A., Mooij, J. M., Heskes, T. & Posthuma, D. MAGMA: Generalized Gene-Set Analysis of GWAS Data. *PLoS Comput. Biol.* **11**, e1004219 (2015).
8. Koopmans, F. *et al.* SynGO: An Evidence-Based, Expert-Curated Knowledge Base for the Synapse. *Neuron* **103**, 217-234.e4 (2019).
9. Benner, C. *et al.* Prospects of Fine-Mapping Trait-Associated Genomic Regions by Using Summary Statistics from Genome-wide Association Studies. *Am. J. Hum. Genet.* **101**, 539–551 (2017).
10. Giambartolomei, C. *et al.* Bayesian Test for Colocalisation between Pairs of Genetic Association Studies Using Summary Statistics. *PLOS Genet.* **10**, e1004383 (2014).
11. Zhao, B. *et al.* Common variants contribute to intrinsic human brain functional networks. *Nat. Genet.* **54**, 508–517 (2022).
12. Tissink, E. *et al.* The Genetic Architectures of Functional and Structural

Connectivity Properties within Cerebral Resting-State Networks. *eNeuro* **10**, (2023).

13. Watanabe, K., Taskesen, E., van Bochoven, A. & Posthuma, D. Functional mapping and annotation of genetic associations with FUMA. *Nat. Commun.* **8**, 1826 (2017).
14. Demontis, D. *et al.* Genome-wide analyses of ADHD identify 27 risk loci, refine the genetic architecture and implicate several cognitive domains. *Nat. Genet.* **55**, 198–208 (2023).
15. Watson, H. J. *et al.* Genome-wide association study identifies eight risk loci and implicates metabo-psychiatric origins for anorexia nervosa. *Nat. Genet.* **51**, 1207–1214 (2019).
16. Wightman, D. P. *et al.* A genome-wide association study with 1,126,563 individuals identifies new risk loci for Alzheimer's disease. *Nat. Genet.* **53**, 1276–1282 (2021).
17. Mullins, N. *et al.* Genome-wide association study of more than 40,000 bipolar disorder cases provides new insights into the underlying biology. *Nat. Genet.* **53**, 817–829 (2021).
18. Howard, D. M. *et al.* Genome-wide meta-analysis of depression identifies 102 independent variants and highlights the importance of the prefrontal brain regions. *Nat. Neurosci.* **22**, 343–352 (2019).
19. Hatoum, A. S. *et al.* Multivariate genome-wide association meta-analysis of over 1 million subjects identifies loci underlying multiple substance use disorders. *Nat. Ment. Health* **1**, 210–223 (2023).
20. Trubetskoy, V. *et al.* Mapping genomic loci implicates genes and synaptic biology in schizophrenia. *Nature* **604**, 502–508 (2022).
21. Xue, A. *et al.* Unravelling the complex causal effects of substance use behaviours on common diseases. *Commun. Med.* **4**, 43 (2024).

**List of Supplementary Note Tables and Figures**

Supplementary Note Figure 1.1. Density plots for non-normal demographic variables. 7

Supplementary Note Table 1.1. Description of the demographic sample and significance of association testing. ........ 8

Supplementary Note Table 1.2. UKB field codes for covariates........ 9

Supplementary Note Table 2.1. Enrichment for different topologies of edges. ........ 10

Supplementary Note Figure 2.1. Raincloud plots for heritability estimates grouped per topology. ........ 11

Supplementary Note Table 3.1. Comparison of SNP-heritability estimates after the application of GSR........ 13

Supplementary Note Figure 3.1. Relationship between measurement reliability and SNP-heritability. ........ 15

Supplementary Note Figure 3.2. Global signal regression (GSR) effect comparison. . 16

Supplementary Note Figure 4.1. Visualisation of locus boundary overlap. ........ 18

Supplementary Note Figure 4.2. Heatmap for $\boldsymbol{H3}$ (left) and $\boldsymbol{H4}$ (right) probability in coloc for all unique pairs of edges associated with the EphA3 locus (*3:89451721:90010903*). ........ 19

Supplementary Note Figure 4.3. Histograms for $\boldsymbol{H4}$ probability in coloc for all unique pairs of edges associated with the *PAX8* (*2:113963070:114213070*), *EphA3* (*3:89451721:90010903*) and *THBS1* locus (*15:39514832:39764832*)........ 19

Supplementary Note Figure 5.1. Comparison of GWS loci with ICA-based phenotype definition. ........ 22

Supplementary Note Figure 5.2. Similarity of edge and RSN genetic architecture. ..... 22

Supplementary Note Table 6.1. Permutation testing results for the effect extent of different neuropsychiatric disorders........ 25

Supplementary Note Figure 6.1. Significant genetic correlations between schizophrenia and FC edges. ........ 26

Supplementary Note Figure 6.2. ADHD GWGAS........ 26

Supplementary Note Figure 6.3. Anorexia GWGAS........ 27

Supplementary Note Figure 6.4. Alzheimer’s Disorder GWGAS........ 27

Supplementary Note Figure 6.5. Bipolar Disorder GWGAS. ........ 27

Supplementary Note Figure 6.6. Depression GWGAS........ 28

Supplementary Note Figure 6.7. Substance Use Disorder GWGAS. ........ 28

Supplementary Note Figure 6.8. Schizophrenia GWGAS. ........ 29

## Supplementary Figures

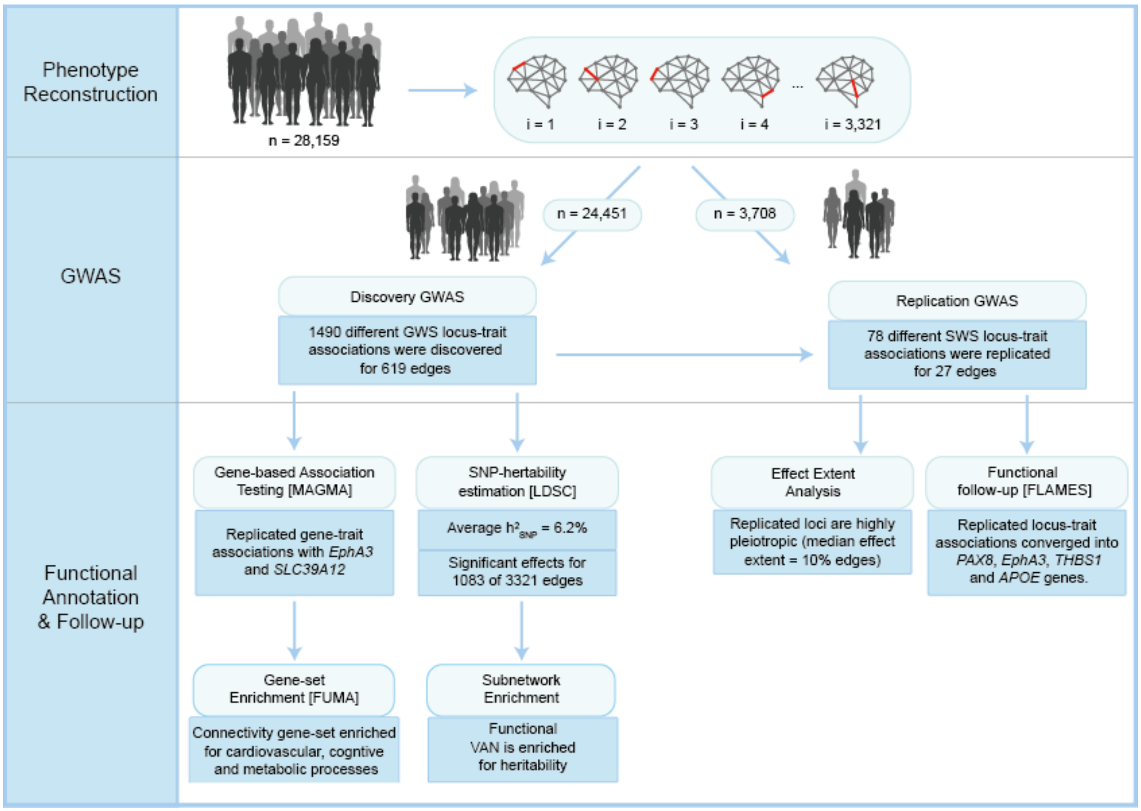


*Supplementary Figure 1. Visual summary of methods and results.*

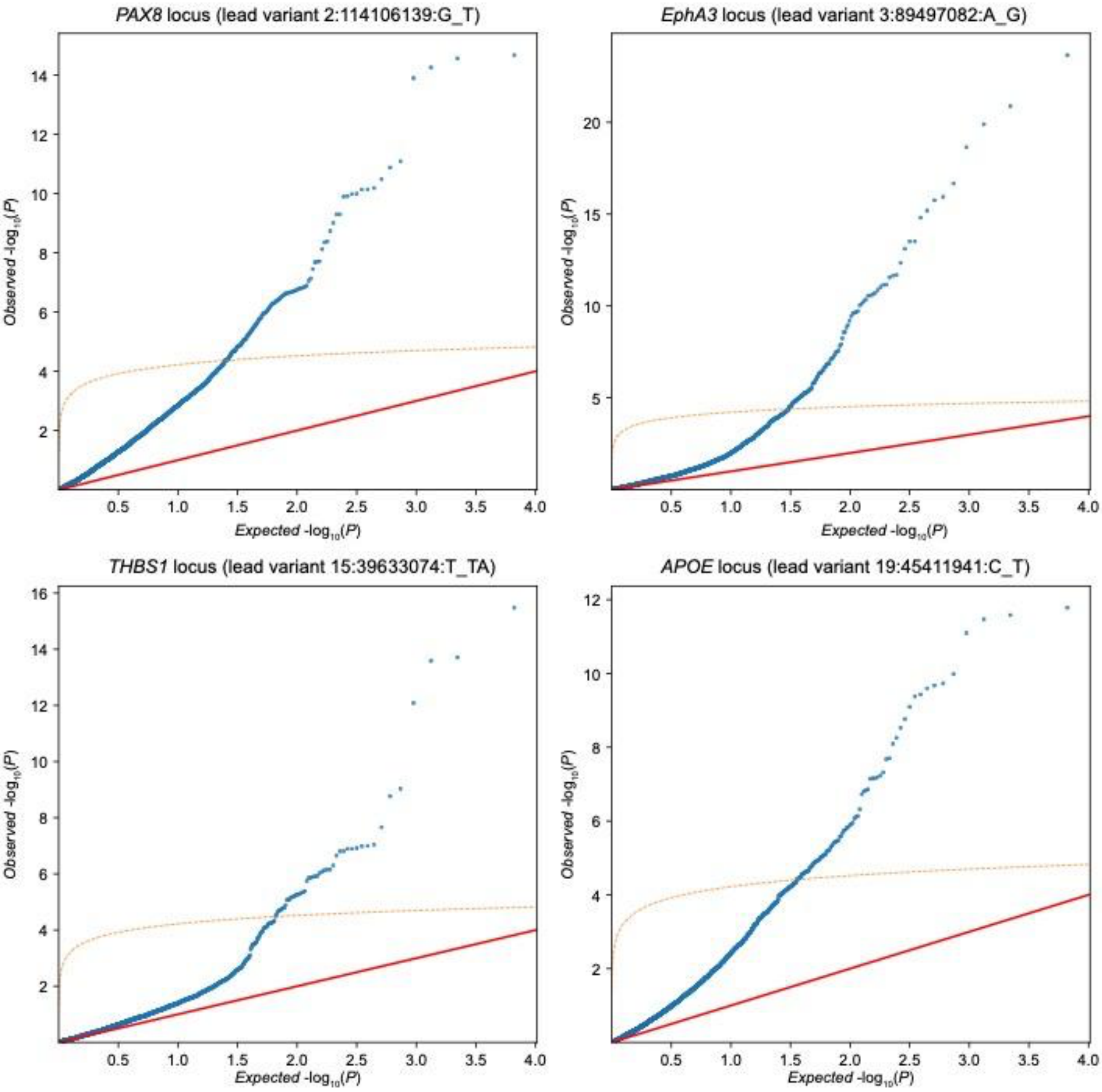


*Supplementary Figure 2. QQ Plots for association of locus across the brain.*

Each point represents one different edge ($N$ = 3321 edges). The red line is the expectation for the p-value distribution under no association. The orange line is the FDR significance level (FDR α= 0.05). The number of points above the orange line is the effect extent of the locus throughout the functional connectome. Only loci study-wide significant ($\alpha_{SWS} = 5\times10^{-8}/3{,}321$) replicated ($\alpha_{rep} = 0.05/208$) loci are represented. *P*-values in QQ plots refer to MAGMA SNP-wise mean locus p-values (Methods).

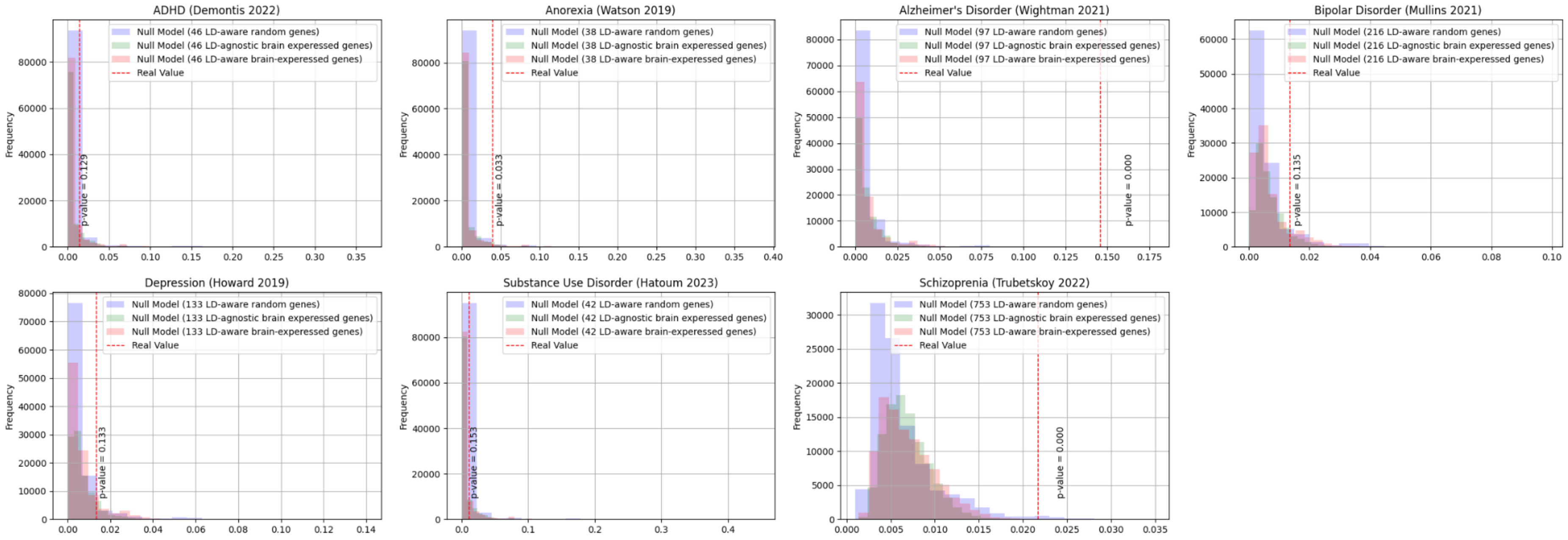


*Supplementary Figure 3. Effect extent of disease genes in the functional connectome.*

Each panel represents a gene-set derived from a different case control disease GWAS. Null-models were built per disorder by drawing (i) a random set of brain genes (green), (ii) a LD-aware set of random genes (blue) or (iii) a LD-aware set of brain genes (red); and calculating its mean effect extent. The number of genes drawn equals the number of significant genes for each disorder: Attention Deficit/Hyperactivity Disorder, Anorexia, Autism Spectrum Disorder, Alzheimer's Disorder, Bipolar Disorder, Depression, Substance Use Disorder, and Schizophrenia. The dashed line is the mean effect extent for the set of disorder genes. *P*-values are calculated based on the null model with an LD-aware set of brain expressed genes. Exact *p*-values are in Supplementary Note Table 6.1.

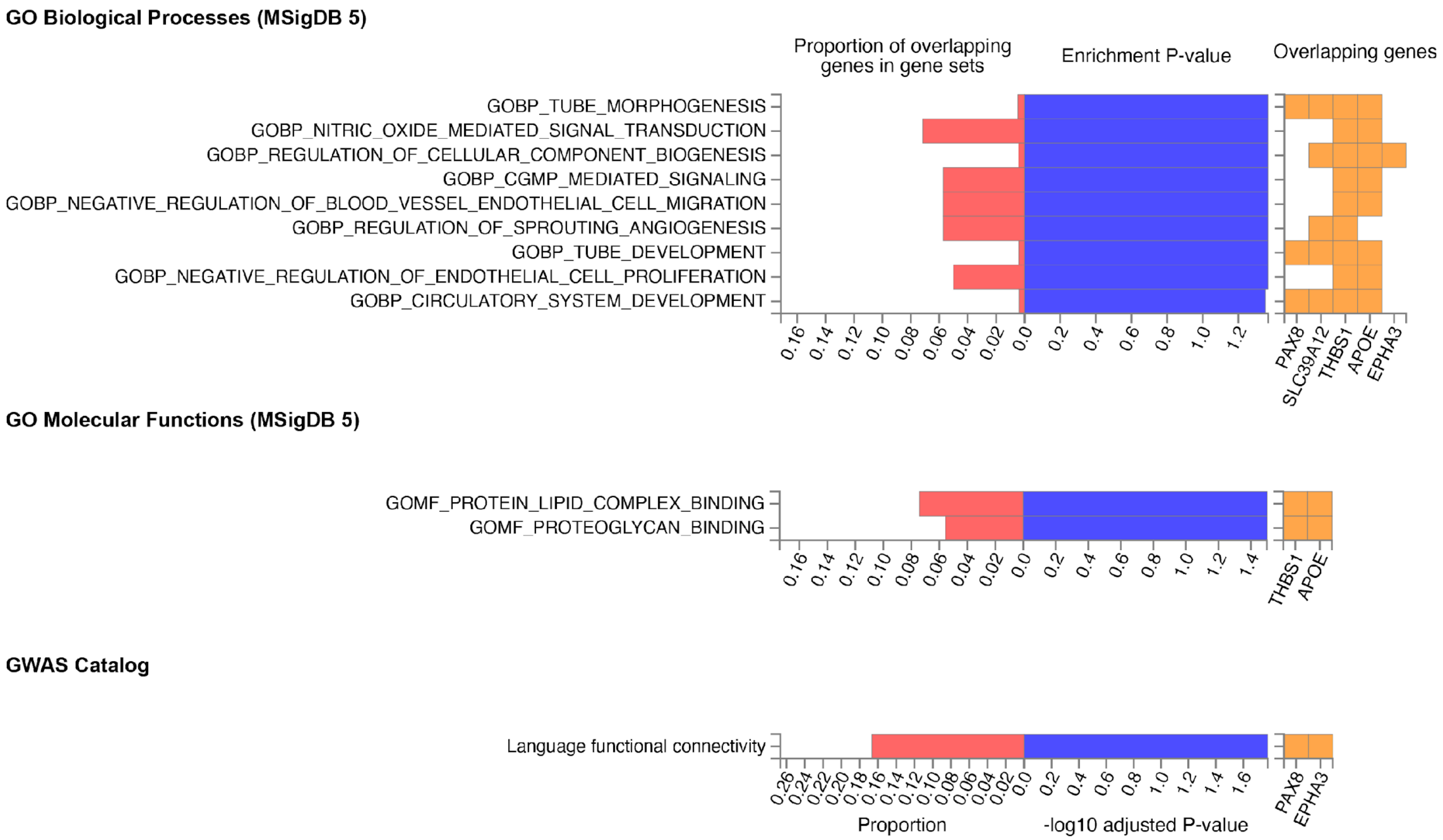


**Supplementary Figure 4. Gene-set enrichment testing for the set of replicated genes (5 genes).**

BH FDR-adjusted *p*-value (one-sided FDR α = 0.05) within each of the three trait categories. Enrichment for Gene Ontology (GO) Biological processes (top), GO Molecular functions (middle) and GWAS catalog (bottom).

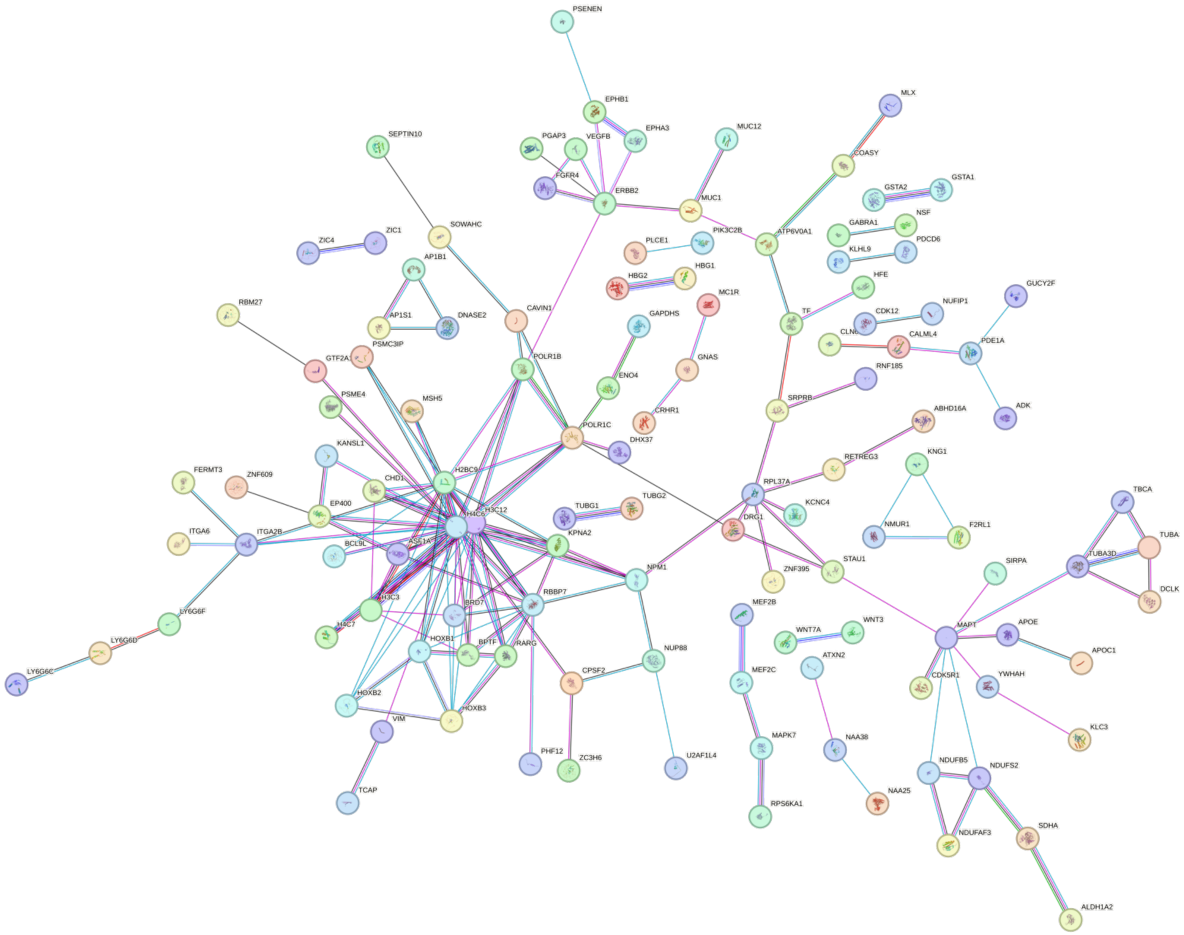


*Supplementary Figure 5. Protein-Protein Interaction Network.*

Evidence is derived from experiments, databases, co-expression, neighbourhood, gene fusion and co-occurrence. Only interacting genes are depicted.

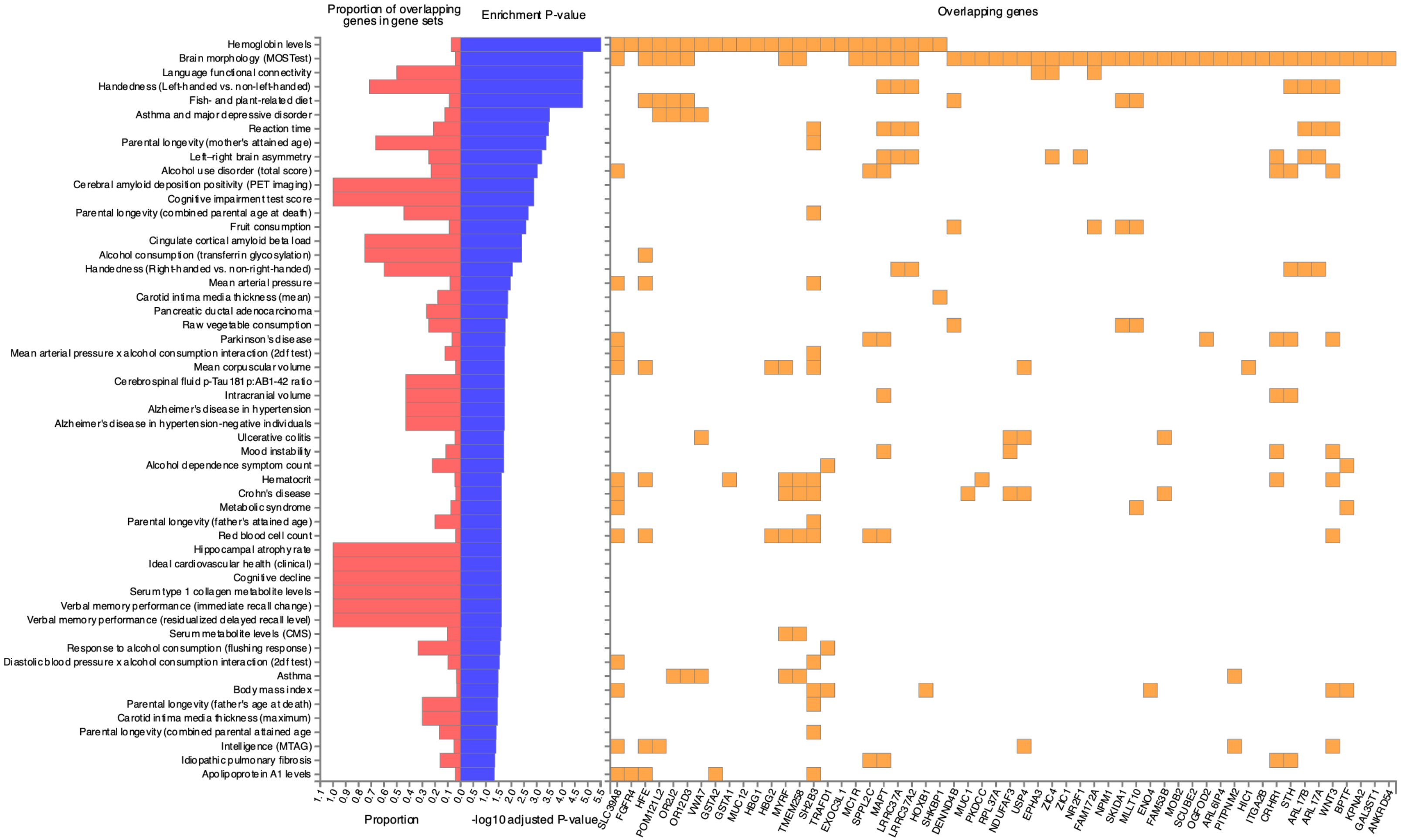


*Supplementary Figure 6. Gene-set enrichment testing for complete set of replicated genes.*

Figure continues in the next page. BH FDR-adjusted *p*-value (one-sided FDR α= 0.05). Right panel represents genes in each gene-set.

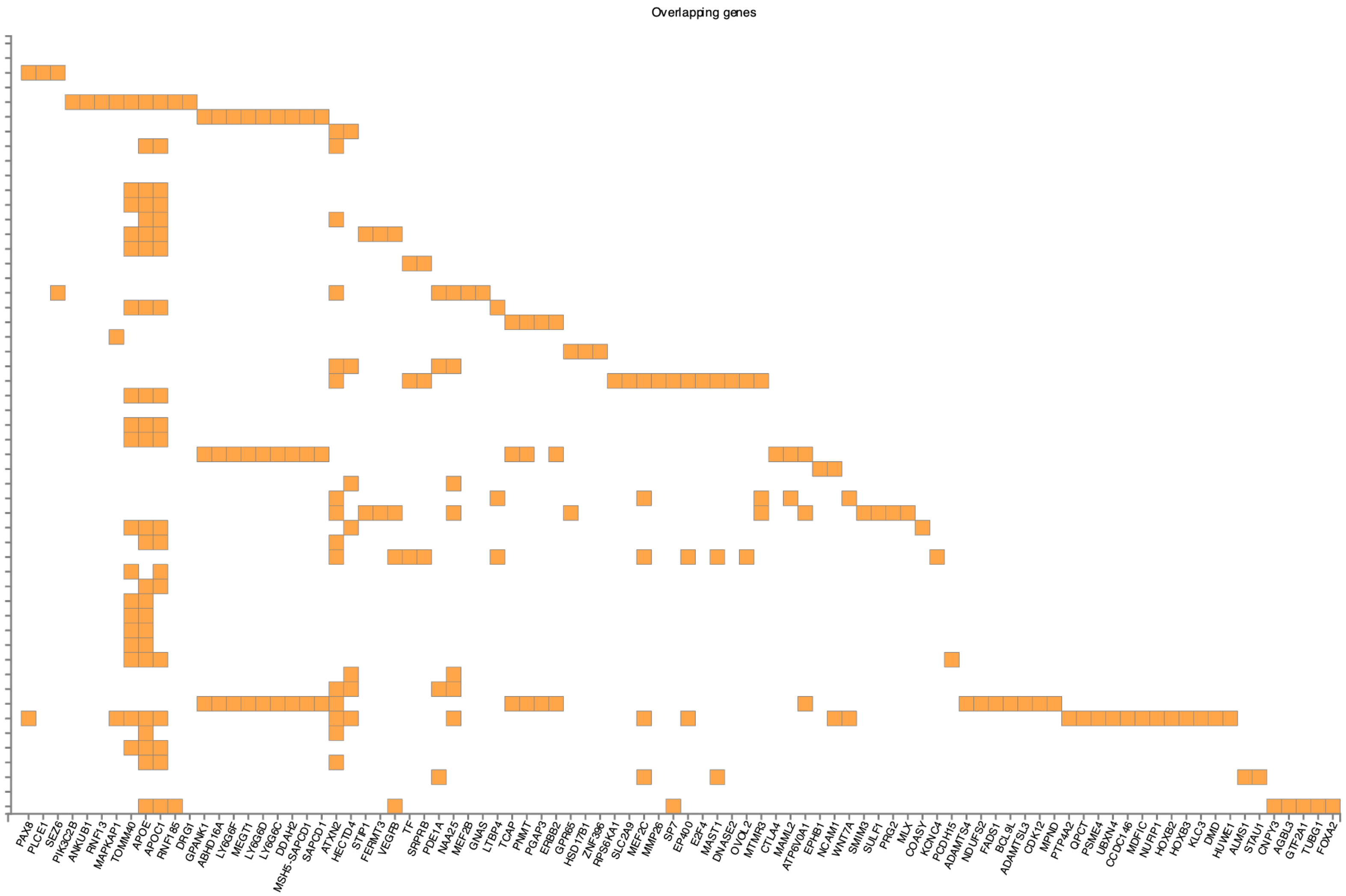


*Supplementary Figure 6. Continued.*

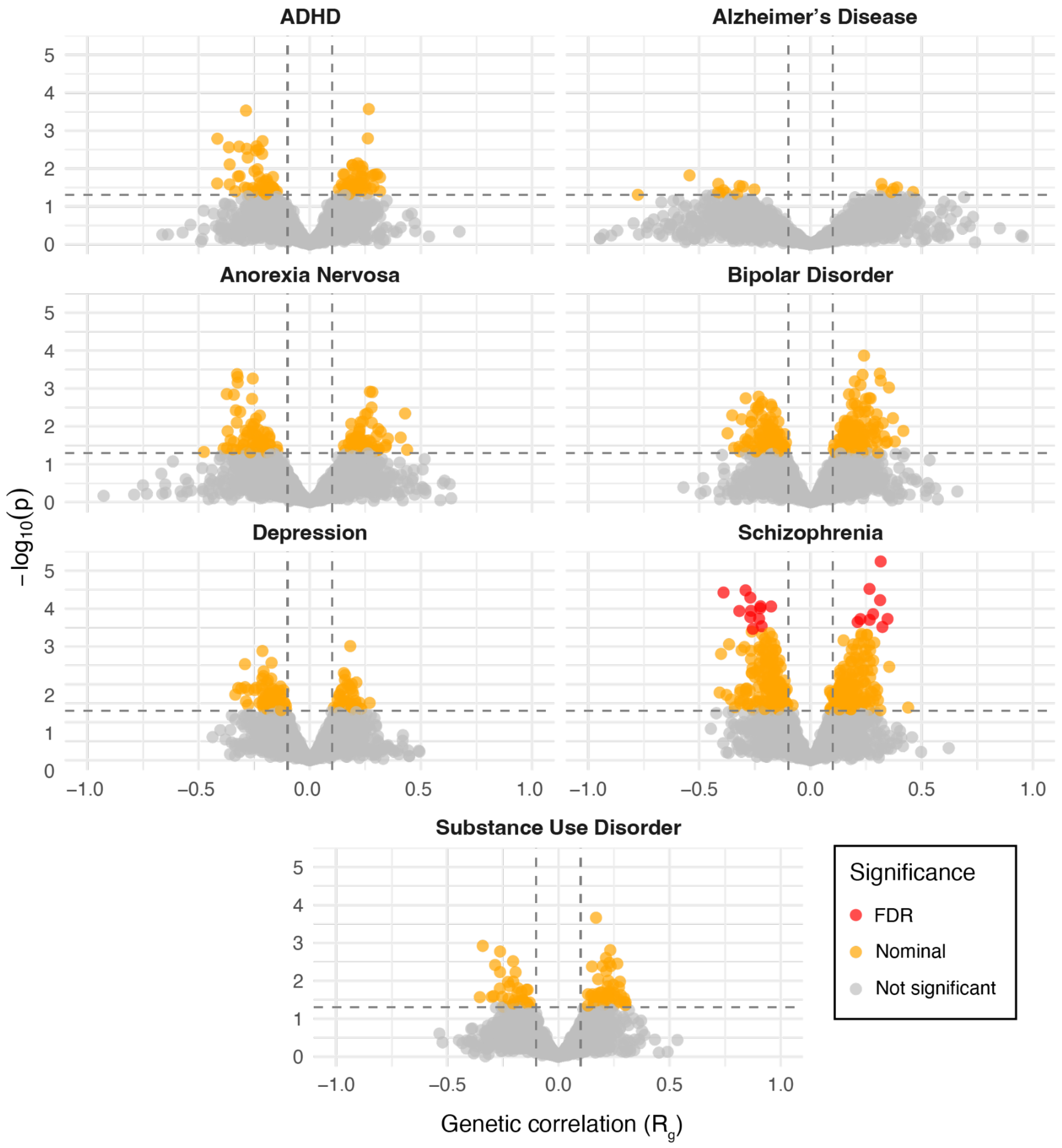


*Supplementary Figure 7. Volcano plots of genetic correlations between edge GWAS and disorder GWAS.*

Each element is a genetic correlation coloured according to its significance (*N* = 3321 edges). In yellow is $p < 0.05$, in red is $q < 0.05$, where *q*-value is the FDR-corrected *p*-value within each disorder across all edges. Each panel refers to the set of genetic correlations of each edge-GWAS with each one of the following GWAS of neuropsychiatric traits: Attention Deficit/Hyperactivity Disorder (ADHD), Alzheimer's Disorder, Anorexia Nervosa, Bipolar Disorder, Depression, Substance Use Disorder, and Schizophrenia

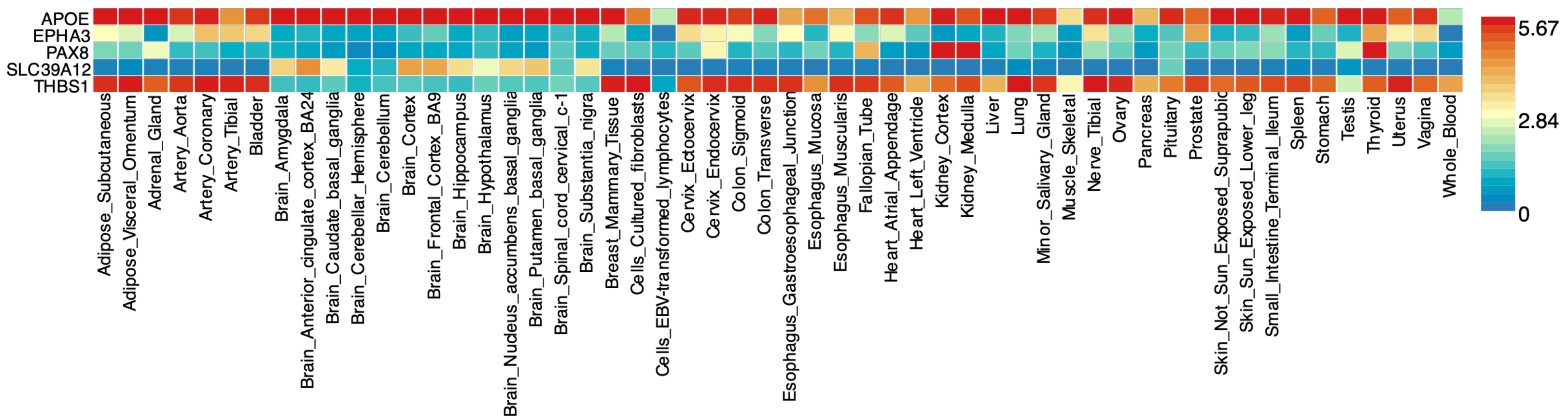


*Supplementary Figure 8. Tissue Expression heatmap of replicated genes.*

Average expression within each tissue ($\log_2$ transformed) is depicted.

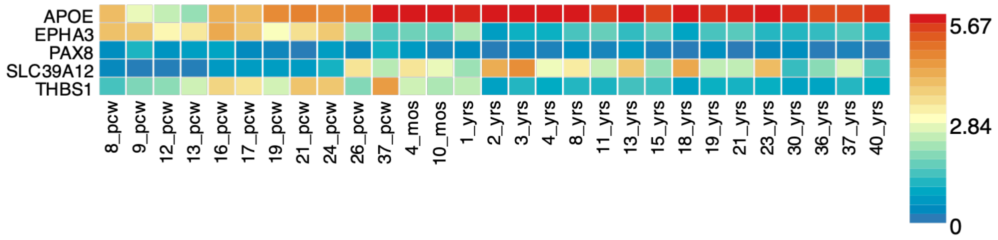


*Supplementary Figure 9. Lifetime expression heatmap of replicated genes.*

Average expression within each life stage ($\log_2$ transformed) is depicted. Pcw: post-conception weeks (prenatal). Yrs: Years (post-natal).
