# Supplementary material for: The genetic landscape of human functional brain connectivity
Source: Nat Commun. 2026 Feb 24;17:3120. doi: 10.1038/s41467-026-69442-9 (PMC13043896; doi:10.1038/s41467-026-69442-9)
Supplement: Supplementary file 2 — Description of Additional Supplementary Files [file 41467_2026_69442_MOESM2_ESM.pdf]

Description of Additional Supplementary Files

File name: Supplementary Data 1
Description: Edgewise SNP-heritability estimation for all 3321 functional edges [LDSC].
Edge: Functional Connection. h2: SNP-heritability Estimate. h2_se: Standard Error of Heritability Estimate. Lambda_GC: genomic inflation coefficient. Intercept: LDSC intercept. Subnetworks: the subnetwork to which each edge belongs. Topology: whether a connection is intrahemispheric. interhemispheric. cortico-subcortical or subcortical. Significant: whether the heritability of an edge was considered significant.

File name: Supplementary Data 2
Description: Replication of SNP-based GWAS results.
LEAD_SNP: SNP ID for lead SNP in the genome-wide significant (GWS) locus in the form chr:pos:a1_a2. where a1 and a2 are alphabetically ordered alleles (GRCh37). CHR: chromosome. X chromosome coded as X and XY, where XY is the pseudoautosomal region of the X chromosome. START: base pair of the most upstream SNP in overlapping locus. END: base pair of the most downstream SNP in overlapping locus. EDGE: the specific edge-GWAS with which the SNP was GWS associated. DISC_P: Uncorrected P-value for lead SNP association with EDGE in the discovery sample. DISC_BETA: linear regression coefficient of LEAD_SNP (genotype) on EDGE (phenotype) in the discovery sample (t-test two-sided alpha = 5e-8/3321) MIN_REP_P: Minimum p-value for the association between all SNPs in locus and EDGE in the replication sample. MEDIAN_REP_BETA: median regression coefficient for the association between all SNPs in locus and EDGE in the replication sample. N_DISC: LEAD_SNP sample size. MAF_DISC: Minor Allele Frequency in discovery sample. REPLICATED: whether the locus was considered discovered with study-wise significance (t-test two-sided discovery alpha = 5e-8/3321) and replicated (t-test one sided replication alpha = 0.05/208 & consistent effect direction).

File name: Supplementary Data 3
Description: Colocalisation results between different edge-GWAS [coloc].
EDGE1. EDGE2: Edges to be colocalised. LOCUS: Genomic Locus of colocalisation in the form chr:start:stop (GRCh37). NSNPs: Number of SNPs used for colocalization H0: posterior probability (PP) of neither trait having a genetic association in LOCUS. H1: PP of only trait 1 having a genetic association in LOCUS. H2: only trait 2 has a genetic association in LOCUS. H3: PP of both traits being associated in LOCUS. but with different causal variants. H4: PP of both traits being associated and sharing a single causal variant in LOCUS.

File name: Supplementary Data 4
Description: Effector gene prediction [FLAMES].
CHR: chromosome. START: base pair of the most upstream SNP in overlapping locus. END: base pair of the most downstream SNP in overlapping locus. EDGE: the specific edge-GWAS with which the SNP was associated. XGB: FLAMES XG Boost Classifier score on functional annotation PoPS: PoPS score for gene in locus. GENE: Gene Symbol. PREDICTED: whether GENE was predicted in the locus for EDGE (FLAMES > 0.05 & Top Prediction for credible set).

File name: Supplementary Data 5
Description: Genetic correlation of individual edges with whole resting-state networks [LDSC].
Tissinketal_RSN: RSN functional connectivity GWAS in Tissink et al. to be correlated with Macieletal_Edge. Macieletal_Edge: Functional Connectivity GWAS in this study genetically correlated with Tissinketal_RSN. SUBNETWORK: Yeo-Krienen RSN that

edge in EDGE_GWAS belongs to. RG: LDSC global genetic correlation estimate (summary statistics with N = 24442 subjects). RG_SE: Standard error of RG estimate. RG_P: Uncorrected P-value of RG. RG_P_FDR: FDR corrected P-value. FDR correction was applied separately for each subnetwork across all edges. BH procedure. (two-sided FDR alpha = 0.05)

File name: Supplementary Data 6
Description: Genetic correlation of individual edges with neuropsychiatric traits [LDSC].
DX_GWAS: Case-control neuropsychiatric GWAS correlated with EDGE_GWAS. EDGE_GWAS: Functional Connectivity GWAS correlated with DX_GWAS. SUBNETWORK: Yeo-Krienen RSN that edge in EDGE_GWAS belongs to. RG: LDSC global genetic correlation estimate (summary statistics with N = 24442 subjects). RG_SE: Standard error of RG estimate. RG_P: Uncorrected P-value of RG. RG_P_FDR: FDR corrected P-value. FDR correction was applied separately for each disorder across all edges. BH procedure. (two-sided FDR alpha = 0.05)

File name: Supplementary Data 7
Description: Summary of discovery gene-based GWAS [MAGMA].
GENE: Gene EntrezID. CHR: chromosome, X chromosome coded as X and XY, where XY is the pseudoautosomal region of the X chromosome. START: Gene start (GRCh37). STOP: Gene stop (GRCh37). NSNPS: Number of SNPs in the gene. N: Sample Size for Association testing. MIN_P: minimum uncorrected P-value for gene-trait association in the discovery sample across all 3321 edges. (Each individual p-value estimated on a discovery sample of N subjects, one-sided alpha = 0.05/18,852) SYMBOL: Gene Symbol

File name: Supplementary Data 8
Description: Summary of replication gene-based GWAS [MAGMA].
GENE: Gene Symbol. EDGE: the specific edge-GWAS with which GENE was associated. START: Gene start (GRCh37). STOP: Gene stop (GRCh37). CHR: chromosome. X chromosome coded as X and XY. where XY is the pseudoautosomal region of the X chromosome. NSNPS: Number of SNPs in the gene. N: Sample size for discovery gene GWGAS. DISC_P: uncorrected P-value for GENE-EDGE association in the discovery sample across all 3321 edges. (Each individual p-value estimated on a discovery sample of N subjects, one-sided discovery alpha = 0.05/(18852x3321))) REP_P: Uncorrected p-value for gene-trait association in the replication sample (N = 3708 subjects for replication, one-sided replication alpha = 0.05/6). REPLICATED: whether the locus was considered replicated (one-sided replication alpha = 0.05/6).

File name: Supplementary Data 9
Description: Gene-set enrichment analyses results (strict gene-set, i.e. SWS replicated genes) [GENE2FUNC]
Category: Database for genesets (one of the category from MsigDB or GWAScatalog). GeneSet : Name of gene set as provided by database. N_genes : Number of genes in a gene set. N_overlap : Number of input genes overlapping with the gene set. p : Hypergeometric test (upper tail) uncorrected one-sided P-value. adjP : BH FDR-Adjusted P-value (one-sided FDR alpha = 0.05 within each trait category). genes : Genes overlapping with the gene set. link : Link to the MsigDB page if available

File name: Supplementary Data 10
Description: Gene-set enrichment analyses results (broad gene-set, i.e. GWS genes) [GENE2FUNC]

Category: Database for genesets (one of the category from MsigDB or GWAScatalog). GeneSet : Name of gene set as provided by database. N_genes : Number of genes in a gene set. N_overlap : Number of input genes overlapping with the gene set. p : Hypergeometric test (upper tail) uncorrected one-sided P-value. adjP : BH FDR-Adjusted P-value (one-sided FDR alpha = 0.05 within each trait category). genes : Genes overlapping with the gene set. link : Link to the MsigDB page if available
