# Supplementary material for: The genetic landscape of human functional brain connectivity
Source: Nat Commun. 2026 Feb 24;17:3120. doi: 10.1038/s41467-026-69442-9 (PMC13043896; doi:10.1038/s41467-026-69442-9)
Supplement: Supplementary file 13 — Reporting Summary [file 41467_2026_69442_MOESM13_ESM.pdf]

nature portfolio

Corresponding author(s): Martijn P. van den Heuvel

Last updated by author(s): Jan 19, 2026

Reporting Summary

Nature Portfolio wishes to improve the reproducibility of the work that we publish. This form provides structure for consistency and transparency in reporting. For further information on Nature Portfolio policies, see our Editorial Policies and the Editorial Policy Checklist.

Statistics

For all statistical analyses, confirm that the following items are present in the figure legend, table legend, main text, or Methods section.

n/a	Confirmed	
☐	☒	The exact sample size (*n*) for each experimental group/condition, given as a discrete number and unit of measurement
☐	☒	A statement on whether measurements were taken from distinct samples or whether the same sample was measured repeatedly
☐	☒	The statistical test(s) used AND whether they are one- or two-sided<br>*Only common tests should be described solely by name; describe more complex techniques in the Methods section.*
☐	☒	A description of all covariates tested
☐	☒	A description of any assumptions or corrections, such as tests of normality and adjustment for multiple comparisons
☐	☒	A full description of the statistical parameters including central tendency (e.g. means) or other basic estimates (e.g. regression coefficient) AND variation (e.g. standard deviation) or associated estimates of uncertainty (e.g. confidence intervals)
☐	☒	For null hypothesis testing, the test statistic (e.g. *F*, *t*, *r*) with confidence intervals, effect sizes, degrees of freedom and *P* value noted<br>*Give P values as exact values whenever suitable.*
☒	☐	For Bayesian analysis, information on the choice of priors and Markov chain Monte Carlo settings
☐	☒	For hierarchical and complex designs, identification of the appropriate level for tests and full reporting of outcomes
☐	☒	Estimates of effect sizes (e.g. Cohen's *d*, Pearson's *r*), indicating how they were calculated

Our web collection on statistics for biologists contains articles on many of the points above.

Software and code

Policy information about availability of computer code

Data collection	n/a
Data analysis	No new software was developed for this project, existing software and code are publicly available.<br>CATO [v3.1.6], http://www.dutchconnectomelab.nl/CATO/;<br>Freesurfer [v6.0]: https://surfer.nmr.mgh.harvard.edu;<br>FUMA, http://fuma.ctglab.nl/;<br>MAGMA [v1.10], https://ctg.cncr.nl/software/magma;<br>LDSC [v.1.0.1], https://github.com/bulik/ldsc;<br>PLINK [v1.9 & v2.0], https://www.cog-genomics.org/plink/;<br>FlashPCA2, https://github.com/gabraham/flashpca;<br>FLAMES [v1.0.0], https://github.com/Marijn-Schipper/FLAMES;<br>FINEMAP [v1.4.1] & LDStore2 [v2.0], http://www.christianbenner.com/;<br>coloc R package [v5.2.2], https://chr1swallace.github.io/coloc/<br>PoPS [v0.2]: https://github.com/FinucaneLab/pops.

For manuscripts utilizing custom algorithms or software that are central to the research but not yet described in published literature, software must be made available to editors and reviewers. We strongly encourage code deposition in a community repository (e.g. GitHub). See the Nature Portfolio guidelines for submitting code & software for further information.

Data

Policy information about availability of data

All manuscripts must include a data availability statement. This statement should provide the following information, where applicable:

- Accession codes, unique identifiers, or web links for publicly available datasets
- A description of any restrictions on data availability
- For clinical datasets or third party data, please ensure that the statement adheres to our policy

The genome-wide summary statistics data generated in this study can be through https://doi.org/10.5281/zenodo.18429460. The imaging and genotyping data are protected and are not available due to data privacy. Source data used to plot each figure are provided with this paper. The following data have been used to perform the analyses on this manuscript: LD reference for LDSC, https://www.internationalgenome.org/category/reference/; RSN Annotation Files, https://surfer.nmr.mgh.harvard.edu/fswiki/CorticalParcellation_Yeo2011; MNI template: https://nist.mni.mcgill.ca/mni-average-brain-305-mri/; FLAMES pathway-naïve feature set: https://zenodo.org/records/10409723; Case-control disease sumstats: https://pgc.unc.edu/for-researchers/download-results/.

Research involving human participants, their data, or biological material

Policy information about studies with human participants or human data. See also policy information about sex, gender (identity/presentation), and sexual orientation and race, ethnicity and racism.

Reporting on sex and gender	Sex in this study reports to an individual's inferred chromosomal sex (i.e. XX-female; XY-male). Subjects with 481 subjects with sex aneuploidy, 370 with discordant reported gender and chromosomal sex were excluded from the analysis as it may indicate poor genotyping quality in the sex chromosomes. Due to the influence of sex in neuroimaging studies (10.1002/hbm.25438), all analyses using subject-level data in this manuscript include inferred chromosomal sex as a covariate.
Reporting on race, ethnicity, or other socially relevant groupings	Only individuals of European ancestry were included in this study. The determination of ancestral groups is necessary to control for population stratification in GWAS. Principal components from the 1000 Genomes reference populations were projected onto the called genotypes available in the UKB data and all 2,034 subjects for whom the projected scores were the furthest to the average score of Europeans (i.e. Mahalanobis distance to the average of Europeans < 6) were excluded from further analysis. The low sample size in the remaining ancestries (<500 individuals) percluded further ancestry specific analyses. The use of a single cohort of individuals of European ancestry — together with sample-specific characteristics like volunteering and ascertainment biases, older age range, and socioeconomic status of subjects — may limit the generalisability of the findings in the present study.
Population characteristics	The full study comprised a discovery and replication sample of 24,451 and 3,708 unique unrelated individuals of European ancestry. 13152 and 2046 individuals were inferred chromosomal females, in discovery and replication, respectively. Median age of participants is 64 years of age. 9.4% of subjects are left-handed.
Recruitment	Participants were invited for an initial in-person visit to a study assessment centre in 2006-2010, during which numerous physical measurements and surveys were administered and blood samples were collected for genotyping. Participant data was linked to medical records via the National Health Services, and a subset of participants also completed MRI scans and additional follow-up data collection in subsequent years. See above for statement on the generalisability of findings given the volunteering bias.
Ethics oversight	The UKB received ethical approval from the National Research Ethics Service Committee North West-Haydock (reference 11/NW/0382), and all study procedures were in accordance with the World Medical Association for medical research. Access to the UK Biobank data was obtained under application number 16406.

Note that full information on the approval of the study protocol must also be provided in the manuscript.

Field-specific reporting

Please select the one below that is the best fit for your research. If you are not sure, read the appropriate sections before making your selection.

☒ Life sciences ☐ Behavioural & social sciences ☐ Ecological, evolutionary & environmental sciences

For a reference copy of the document with all sections, see nature.com/documents/nr-reporting-summary-flat.pdf

Life sciences study design

All studies must disclose on these points even when the disclosure is negative.

Sample size	Data were derived from the UK Biobank (UKB), a population-based cohort sample of approximately 500,000 adults in the UK. Participants were invited for an initial in-person visit to a study assessment centre in 2006-2010, during which numerous physical measurements and surveys were administered and blood samples were collected for genotyping. All available unrelated subjects with one imaging visit available (n = 36,969) subjects with were considered to this manuscript. Data exlusions were performed as decribed below.
Data exclusions	In the full UKB sample, 481 subjects with sex aneuploidy, 370 with discordant reported and chromosomal sex, and 195 with high degrees of relatedness were excluded from further analyses. Next, on the subset of subjects with one imaging visit available (n = 36,969), related subjects were excluded. Subjects with high levels of kinship (KING coefficient>0.4) and the most inferred relatives were removed until no two subjects were reported to be third-degree relatives (or closer), accounting for a total of 5,241 exclusions. Population stratification was controlled by

	correcting for principal genomic components calculated with FLASHPCA2.1 Principal components from the 1000 Genomes reference populations2 were projected onto the called genotypes available in the UKB data and all 2,034 subjects for whom the projected scores were the furthest to the average score of Europeans (i.e. Mahalanobis distance to the average of Europeans < 6) were excluded from further analysis. Finally, 1,341 subjects considered to be imaging outliers were excluded from the analysis. These were subjects for which average head motion parameters, signal-to-noise ratio or discrepancy between T1-weighted and fMRI scan deviated from the median more than five times their median absolute variation.3 These genotyping data were collected with two different array types - UK Biobank Axiom (UKBA) and the UK BiLEVE (UKBB) arrays - which cover 812,428 unique genetic markers and overlap 95% in SNP content.
Replication	A hold-out set of 5000 randomly selected unrelated individual was allocated prior to the analyses. Gene and SNP findings were submitted to replication. Out of the 208 SNP-based assocations, 78 replicated. Out of the 6 found gene-edge associations, 2 replicated. Where replication was possible, only successfully replicated results were discussed further, non-replicated results are clearly noted in the text.
Randomization	Allocation to the replication sample was performed by creating a randomised hold-out set of 5000 individuals prior to outlier removal and data exclusion as described above.
Blinding	Data from a biobank was analysed by this group of researchers with no involvement in data collection. Blinding is not applicable to this single group observational study.

Reporting for specific materials, systems and methods

We require information from authors about some types of materials, experimental systems and methods used in many studies. Here, indicate whether each material, system or method listed is relevant to your study. If you are not sure if a list item applies to your research, read the appropriate section before selecting a response.

Materials & experimental systems

n/a	Involved in the study
☒	☐ Antibodies
☒	☐ Eukaryotic cell lines
☒	☐ Palaeontology and archaeology
☒	☐ Animals and other organisms
☒	☐ Clinical data
☒	☐ Dual use research of concern
☒	☐ Plants

Methods

n/a	Involved in the study
☒	☐ ChIP-seq
☒	☐ Flow cytometry
☐	☒ MRI-based neuroimaging

Plants

Seed stocks	*Report on the source of all seed stocks or other plant material used. If applicable, state the seed stock centre and catalogue number. If plant specimens were collected from the field, describe the collection location, date and sampling procedures.*
Novel plant genotypes	*Describe the methods by which all novel plant genotypes were produced. This includes those generated by transgenic approaches, gene editing, chemical/radiation-based mutagenesis and hybridization. For transgenic lines, describe the transformation method, the number of independent lines analyzed and the generation upon which experiments were performed. For gene-edited lines, describe the editor used, the endogenous sequence targeted for editing, the targeting guide RNA sequence (if applicable) and how the editor was applied.*
Authentication	*Describe any authentication procedures for each seed stock used or novel genotype generated. Describe any experiments used to assess the effect of a mutation and, where applicable, how potential secondary effects (e.g. second site T-DNA insertions, mosiacism, off-target gene editing) were examined.*

Magnetic resonance imaging

Experimental design

Design type	Resting-state functional MRI
Design specifications	n/a
Behavioral performance measures	n/a

Acquisition

Imaging type(s)	Functional
Field strength	3T
Sequence & imaging parameters	Resolution: 2.4x2.4x2.4 mm<br>Field-of-view: 88x88x64 matrix<br>Duration: 6 minutes (490 timepoints)

TR: 0.735 s
TE: 39ms
GE-EPI with x8 multislice acceleration, no iPAT, flip angle 52deg, fat saturation

Area of acquisition: Whole-brain

Diffusion MRI: ☐ Used ☒ Not used

Preprocessing

Preprocessing software: Functional connectivity was computed using CATO (Connectivity Analysis Toolbox; v3.1.6). Resting-state functional magnetic imaging was used with T1 surface model files and structural segmentation from FreeSurfer (v6.0).

Normalization: Coregistration was performed by aligning the subject average of rs-fMRI across all time points with the T1 image.

Normalization template: Data were processed in the native (subject) space.

Noise and artifact removal: Motion metrics, alongside their first-order drifts and their linear trends, and the average signal of voxels in both cerebrospinal fluid and white matter were regressed out of the rs-fMRI signal. A zero-lag bandpass filter ([0.01-0.1] Hz band) was applied.

Volume censoring: Motion-scrubbing were applied to the time series (max FD = 0.25, max DVARS = 1.5, min violations = 2, backward neighbours = 1, forward neighbours = 0).

Statistical modeling & inference

Model type and settings: Genome-wide association studies of functional connectivity strength. Identification of common genetic variants involved in functional connectivity was carried out using PLINK2.0. A total of 3,321 SNP-based GWAS on single-edge connectomic data were performed for 8,790,386 imputed and genotyped SNPs. Phenotypes were standardised across subjects and residualised for total intracranial volume. This analysis was performed on independent (Linkage Disequilibrium threshold, r2>0.1), common (MAF > 0.1), and genotyped SNPs or SNPs with very high imputation quality (INFO>0.9). The first 20 principal components were used as covariates in all GWAS together with sex, age, handedness, genotype array type, and covariates specific to fMRI: scanning site, time to echo, table coordinates, coil position, signal-to-noise ratio, mean head motion, intensity scaling parameters and framewise displacement. The full set of covariates was standardised. Male X variants were coded as 0/1 (assuming no deactivation) to avoid double counting.

Effect(s) tested: Testing SNP effects on average functional connectivity.

Specify type of analysis: ☐ Whole brain ☒ ROI-based ☐ Both

Anatomical location(s): Surface-based cortical parcellation was performed using FreeSurfer, parcellating the cortical mantle according to the Desikan-Kiliany atlas.74 Subcortical volumes were automatically segmented into the aseg atlas in the T1 image.75 This resulted in a whole brain parcellation of a total of 82 brain areas (68 cortical + 14 subcortical)

Statistic type for inference (See Eklund et al. 2016): n/a

Correction: n/a. In the genetic analyses, bonferroni correction for the number of independent genetic variants and number of edges was adopted.

Models & analysis

n/a	Involved in the study
☐	☒ Functional and/or effective connectivity
☒	☐ Graph analysis
☒	☐ Multivariate modeling or predictive analysis

Functional and/or effective connectivity: Functional connectivity was constructed by calculating pairwise Pearson's correlations between the average preprocessed time series of each of the 82 parcellated brain areas.
